# Rural food markets and their implications for the availability and affordability of nutrient-dense foods in Bihar, India

**DOI:** 10.1371/journal.pone.0361231

**Published:** 2026-10-30

**Authors:** Anjali Purushotham, Bhavani Shankar, Gregory S. Cooper, Martin Watts, Emily Fivian, Helen Harris-Fry, Sai Ram Challa, Suneetha Kadiyala

**Affiliations:** 1 Institute for Sustainable Food and School of Geography and Planning, University of Sheffield, United Kingdom; 2 Department of Population Health, Faculty of Epidemiology and Population Health, London School of Hygiene and Tropical Medicine, London, United Kingdom; 3 Public Health Nutrition Division, Indian Council of Medical Research National Institute of Nutrition, Hyderabad, India; Vivekananda Global University, INDIA

## Abstract

The adequate consumption of nutrient-dense foods such as fruits, vegetables, and animal-source foods is crucial for achieving good health. However, in many low- and middle-income countries (LMICs), consumption of nutrient-dense foods is extremely low. Food markets are a critical node in the rural food environments of LMICs. Yet, they remain understudied and there is little granular information available on how well-equipped they are to supply affordable nutrient-dense foods. We address this gap in knowledge by contributing information on rural food markets from Bihar, India. We use a primary market survey conducted in two districts of Bihar, and examine how market infrastructure, governance, and spatial organisation shape the availability and prices of nutrient-dense foods. By applying agglomerative hierarchical clustering, we identify three market types and link the market typology with information from a separate primary survey of food prices to ask whether prices of nutrient-dense foods differ by market type. There are three main findings of our study: (i) There is a near universal and extreme lack of market infrastructure relevant to making safe and affordable nutrient-dense foods available. (ii) Market types differ largely by size and proximity to the nearest urban centre but display remarkably little difference in infrastructure and governance. (iii) Availability of nutrient-dense foods is lower in remote rural markets, but prices in these markets are not consistently higher than in larger, more urban markets. Our findings highlight that modest infrastructure investments, standardised management practices, and promotion of a diverse mix of long and short supply chains could enhance access to nutrient-dense foods to address nutrition challenges in similar contexts of rural LMICs.

## 1. Introduction

Nutrient-dense foods, particularly fruits, vegetables, and animal source foods, play a crucial role in nutrition and diet-related health outcomes [1,2]. However, significant shares of populations in many low- and middle-income countries (LMICs) consume insufficient quantities of these foods resulting in poor diet quality and micronutrient deficiencies [2,3]. Rural populations in LMICs face particular challenges in consuming nutrient-adequate diets owing to constraints in availability, access, and affordability of nutrient-dense foods [4].

Recent research has linked food environments to dietary and nutrition outcomes in LMICs [5,6]. Traditional food markets in rural areas are primarily retail establishments wherein local farmers and traders sell food items to local consumers. Such markets are a critical component of rural food environments in LMICs [7,8], either as outlets for direct purchase by local consumers, or as hubs from which other outlets, such as village shops and itinerant vendors, draw their supply. Evidence from 45 LMICs shows that food consumption is predominantly market-sourced, even amongst poor and subsistence-oriented farming households [9].

Rural food markets vary widely in size, governance, infrastructure and degrees of informality. Many face persistent constraints limiting their capacity to enable access to nutrient-dense foods. Most fruits, vegetables, and animal-source foods are highly perishable, and yet there is typically very limited market-level cold, or adequate non-cold storage, increasing risk of food losses in comparison to well-equipped formal market establishments. Unsafe food is often a concern in rural food markets, and has been linked to the spread of pathogens, contaminants, and, in rare cases, zoonotic disease [10,11]. Infrastructural inadequacies also mean that produce is increasingly exposed to extreme heat and floods arising from climate change [12], in turn worsening food loss and safety. Inadequate transport and communication infrastructure between food markets may mean that nutrient-dense foods cannot move efficiently from surplus to deficit areas, contributing to localised shortages and high prices in more remote settings [13]. Together, these constraints are likely to be more acute in smaller, more informal markets, resulting in important implications for both the availability and prices of nutrient-dense foods.

However, despite the importance of traditional food markets in nutrient-dense food access and the constraints they face in realising their potential, there is remarkably little granular evidence on the state of food markets in LMICs and their implications for the availability and prices of nutrient-dense foods to rural populations. Reflecting rapid urbanisation and associated dietary shifts towards convenient but unhealthy ultra-processed foods [14], research to date has largely prioritised urban food environments often overlooking rural markets that remain the primary source of nutrient-dense foods for rural populations [6,15,16].

Emerging policy frameworks such as the concepts of rural “Territorial Markets” [17] and “Safer Traditional Food Markets” [18] increasingly recognise the potential of traditional food markets to support healthy diet. Yet, currently, there are limited empirical benchmarks to assess how rural food markets compare to good-practice standards in hygiene, safety, storage, and connectivity, or to identify where the targeted investments could have the greatest nutritional returns [19].

To fill this gap, this paper provides new evidence using two unique market-level primary surveys from Bihar, India. Using Bihar as a case of a predominantly rural and low-income food system, we address the following research questions: (i) How well-equipped are rural traditional food markets in terms of infrastructure, facilities, and connectivity relevant to making safe and affordable nutrient-dense foods available? (ii) What are the broad types of rural food markets, and what are the main ways in which they differ from one another? (iii) Do the availability and prices of nutrient-dense foods vary systematically by market type?

## 2. Conceptual framework

Fig 1 presents a conceptual framework depicting how market infrastructure and facilities, the central components of this study, is linked with market food environments and thereon to higher level outcomes relating to food consumption, diets and nutrition. It builds upon the existing conceptual framework by [20] that connects food environments to consumption and dietary outcomes. We consider four categories of market infrastructure: (i) *hard infrastructure* encompassing aspects such as raised platforms, roof, drainage and toilets; (ii) *storage infrastructure* that includes both market-level cold storage as well as basic storage materials such as ventilated baskets for vendors and room for keeping spare produce protected from weather; (iii) *utilities* such as electricity and water supply and waste removal services; (iv) *connective infrastructure* that links markets and helps produce flow smoothly between them. The link from geography and endowments to market infrastructure hypothesises that the types of infrastructure found in markets may depend on urban-rural gradients and local economic development, which is an aspect descriptively examined in this study.

**Fig 1:**
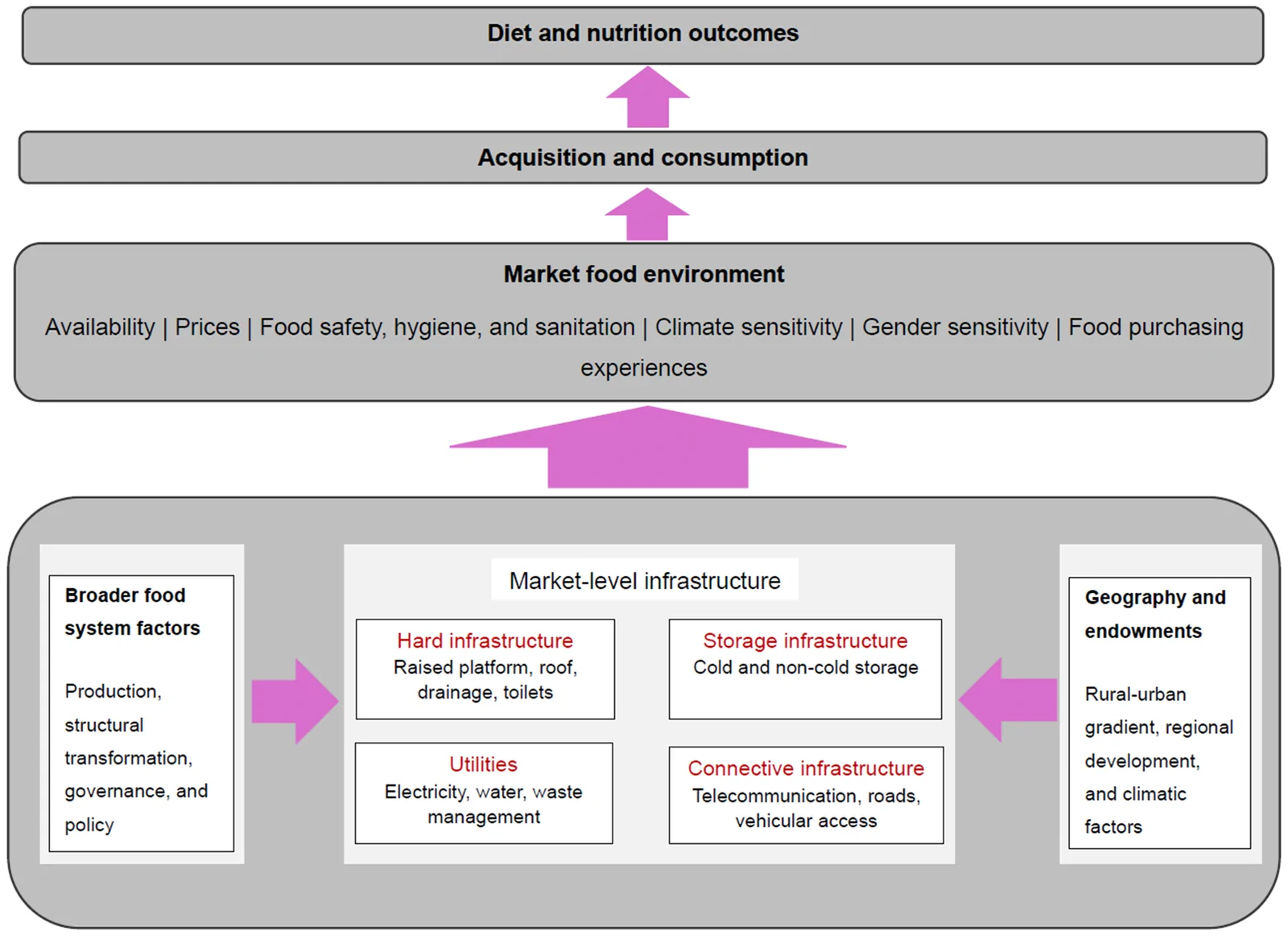
Conceptual framework linking market infrastructure with market food environment and dietary outcomes.

Market infrastructure, geography and endowments, along with a set of broader food systems factors is shown to be associated with market-level food environments [21]. Market food environments include diverse aspects such as food availability and prices, food safety, hygiene and sanitation, climate and gender sensitivity at the market, and overall food purchasing experience. A single arrow is shown linking market infrastructural components to market food environment aspects; however, this is an aggregate representation of several specific pathways. For example, raised platforms, water supply, waste and drainage management have a key role in food safety, sanitation, whereas roof and storage infrastructure are particularly important for climate sensitivity. Several infrastructure types may be of relevance to availability and prices, especially for perishable nutrient-dense foods. For example, availability of storage may encourage inward flow of produce and improve availability by lowering market-level food loss. Whereas better connective infrastructure may encourage cross-market flows and therefore influence availability and prices. Remoteness and urban proximity may be relevant to availability and prices not only through their influence on infrastructural availability at the market, but also independently. For example, urban proximity can offer economies of scale in flows into markets, and remoteness raises transport costs even when mitigated by good connective infrastructure, with potential implications for availability and prices [22]. Fig 1 summarises these components and pathways as a guidance framework for the descriptive exploration of market infrastructure in our study.

## 3. Study area, sampling, and data collection

### Study setting

Our study is set in Bihar (Fig 2), India’s third-most populous state (>104 million) and the most multidimensionally poor [23]. A vast number of rural traditional food markets (also known as *‘haats’*), are spread throughout the state and are a key retail node for nutrient-dense foods. As such, Bihar provides a particularly salient setting to examine the role of rural food markets in shaping access to nutrient-dense foods.

**Fig 2:**
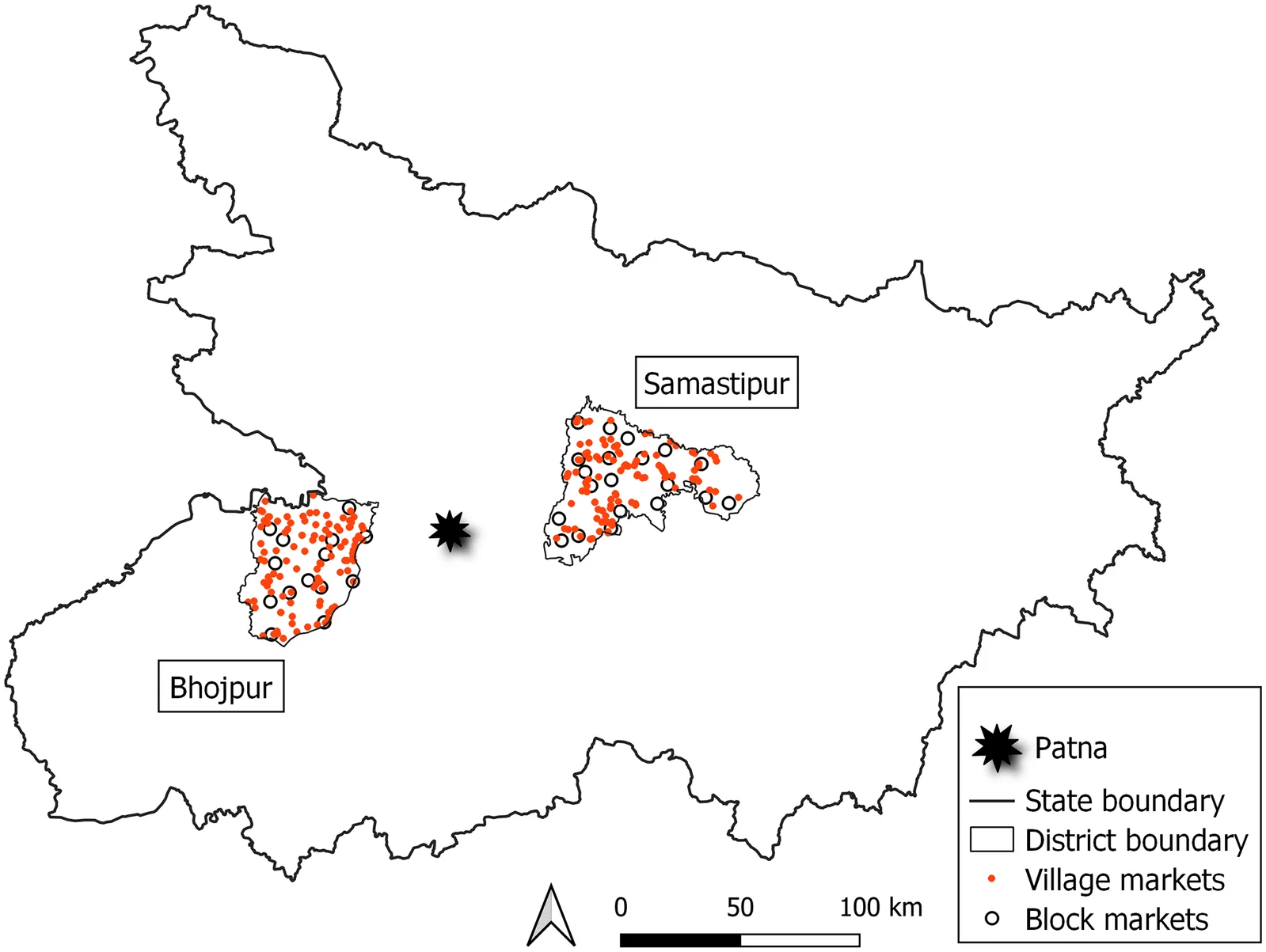
Study area, sample districts, and sample markets (Map source: The location coordinates for the village and gram panchayat (GP) markets are collected from the primary survey in Bihar and the rest of the information presented in the map is taken from Dataset of Global Administrative Areas (GADM)).

Agriculture is the primary livelihood for over 75 percent of Bihar’s population, most of whom are marginal landholders. The state is among the major producers of fruits and vegetables in the country [24]. Despite the strong agrarian base and production of nutrient-dense foods, diet quality remains dismally poor [25]. This is reflected in the poor nutritional outcomes among different demographic groups – a 42 percent stunting (low height-for-age) prevalence among children below five years, and a 63 percent anaemia prevalence among women aged 15–49 years [26].

Unlike other Indian states, Bihar does not operate under the Agricultural Produce Market Committee (APMC) Act that states specific guidelines and standards for the regulated wholesale marketing of agricultural commodities, having withdrawn from the Act in 2006 [27]. As a result, the state lacks a formalised regulatory and governance framework overseeing agricultural and food marketing activities. While national datasets such as Mission Antyodaya provide village level information on aspects such as distance to the nearest food market, they offer little insight on the physical condition, scale, governance arrangements, or the functional capacity and the availability or the prices of nutrient-dense foods in these markets [28]. In this context, Bihar presents a critical case for understanding how traditional food market systems function in predominantly rural and low-income settings of LMICs, where formal market regulation is limited, yet play a critical role in access to nutrient-dense foods.

### Sampling

We collected traditional food market data from two districts in Bihar – Bhojpur and Samastipur – located southwest and northeast of Bihar’s capital, Patna, respectively (Fig 2). These districts were selected in consultation with the Bihar State Livelihood Mission (JEEViKA). They are among the priority districts for the development initiatives of the Government of Bihar and enable geographical diversity in the sampling of markets.

We used a stratified random sampling approach to sample 227 rural markets. This was conducted in four steps. First, all “Gram Panchayats (GPs)” (administrative units comprising groups of villages) in each district were mapped. As per the latest available Mission Antyodaya data [28], there were 228 and 381 GPs in Bhojpur and Samastipur, respectively. These GPs served as the primary sampling units. Second, the Euclidean distance from each GP centre to the nearest Block (administrative layer between the GP level and the district level) headquarters was calculated. The distance to the nearest Block headquarters was used as a proxy for market access and economic development [22,29], with GPs located close to Block headquarters assumed to have better access to all-weather roads, markets, and permanent shops that operate daily and sell a variety of nutrient-dense foods. In the third step, the distance to the nearest block headquarters was categorised into six strata, with GPs in stratum 1 having the highest and the GPs in stratum 6 having the lowest distances to the block headquarters. In each stratum, 12–15 GPs were randomly selected proportional to the size of strata (165 GPs in total, 91 in Bhojpur and 74 in Samastipur). Finally, in each of the selected GPs, all existing food markets were sampled, resulting in a sample of 227 markets from the two districts (109 and 118 markets in Bhojpur and Samastipur, respectively). In addition, we also sampled 28 larger block-level markets that play an important role in supplying food items to the rural food markets sampled at the GP level. There are 34 blocks in the two study districts, of which 6 block markets were covered in the sampling strategy adopted to select the rural markets at the GP level. To select the remaining markets in 28 blocks, we first mapped all the markets within each block and stratified them by their size (i.e., number of vendors operating in the markets). Then the largest markets within the size strata were selected for inclusion in our sample, thus giving a total of 255 markets.

### Data collection

In each of the sampled 255 markets, we conducted structured surveys with market managers or experienced vendors in the market and carried out structured market observations during July 2024. Written consent was obtained from the respondents of the structured market surveys. Following the World Health Organisation (WHO) guides for healthy food markets [30], we collected information on a range of market functions, governance, infrastructure, and accessibility factors. In addition, following the established food environment approaches that assess food availability [31,32], we collected information on the number of vendors selling different nutrient-dense food groups.

In these same markets, we also collected price information for seventeen nutrient-dense foods frequently consumed in the region during July-August 2024. These include tomato, brinjal (aubergine), cabbage, cauliflower, spinach, sponge gourd, banana, grapes, mango, papaya, jackfruit, egg, painasa fish, chicken (broiler variety), and chicken (desi (local) variety). These foods were chosen to represent a diversity of nutrient-dense food types and their importance in local diets, as indicated by pilot data collection and secondary sources [33]. Information was also collected on potato and onion, which are heavily traded and widely consumed foods in Bihar and serve as comparators for the other foods included in the price survey. The methodology for collecting price data included a transect walk of the market to note if and how many vendors were selling one or more of the listed 17 nutrient-dense foods in the market. This was followed by selecting a maximum of three vendors offering the largest selection of the targeted 17 nutrient-dense foods to collect price information. To ensure prices are collected for broadly comparable and edible quality foods commonly sold in the market, only foods that are not visibly rotten or damaged and are offered in large quantities by the selected vendors were chosen to collect the price information. Verbal consent was obtained from the vendors selected for the price data collection. Price information was collected on every market day, except in markets operating more than twice a week – in those cases, prices were collected twice weekly. Any personal identifiers were anonymized before using the curated data for statistical analyses.

Ethical approval was obtained from relevant Institutional Review Boards (the University of Sheffield (reference number: 060310, date: 20.05.2024), the London School of Hygiene and Tropical Medicine (reference number: 31001, date: 07.06.2024), ICMR National Institute of Nutrition: Institutional Ethical Committee (reference number: CR/4/III/2023, date: 03.10.2023), and DIA Evidence and Impact (reference number BR-INF-01/2024–2025, date: 10.06.2024)), and survey tools and procedures were piloted in nearby markets outside our sample.

## 4. Data analyses

We employ descriptive statistics and data visualisation tools to address the first research question on how well-equipped traditional rural food markets are in facilitating availability of safe nutrient-dense foods. We descriptively analyse market functions, governance, infrastructure, and accessibility characteristics, and discuss their role as key nodes of the rural food systems in Bihar. We do this by presenting summary statistics and group-wise comparisons. Since the Shapiro-Wilk test within each comparison group violated the normality assumption for one or more groups across the continuous variables included in the analyses, we use non-parametric tests such as the Mann-Whitney U test for two-group comparisons and Kruskal-Wallis test for comparisons across more than two-group. In addition, Pearson’s Chi^2^ test is used for comparison of factor variables across groups.

We then conduct an agglomerative hierarchical cluster analysis to identify whether distinct market types exist in our sample, addressing the second research question. Hierarchical clustering follows a bottom-up approach, which first groups the most similar market characteristics and successively merges markets based on the smallest difference between market characteristics until a single cluster remains [34]. For the cluster analysis, we include all the market functional, governance, infrastructure, and accessibility characteristics described in the previous descriptive analysis. Since these characteristics include both continuous and factorial responses in the survey, hierarchical clustering is most appropriate as it employs Gower’s distance matrix, which combines appropriate distance measures for each variable type into a single dissimilarity score [35]. Following this, we use Ward’s agglomerative linkage method to the resulting distance matrix, which helps to construct a dendrogram and identify the distinct market types with minimum intra-cluster variance [36]. The optimal number of clusters are identified through a visual inspection of dendrogram and the average silhouette width across alternative cluster numbers.

To study the third research question – do the availability and prices of nutrient-dense foods vary by market type? – we conduct further descriptive and exploratory analyses by market types identified using cluster analysis. First, to proxy the availability of nutrient-dense foods in the sample markets, we calculate the number of vendors selling each of six different nutrient-dense food groups: vegetables, fruits, dairy, eggs, fish, and meat. Second, we use the price per unit of the 17 frequently consumed nutrient-dense foods noted above to calculate a market-level price for each nutrient-dense food. We compute market-level prices as the average across vendors for each nutrient-dense food. In instances where specific items were unavailable in certain sample markets on data collection days, the average reflects the prices recorded on the days they were available. By comparing averages across market types as revealed by the cluster analysis, we aim to understand if there are any differences in the availability and prices of nutrient-dense foods given the status of operation, infrastructure facilities, and governance of market types.

All data curation and analyses presented in the study were done using statistical tools STATA 19.5 and R Studio.

## 5. Results

### 5.1. Market characteristics

Table 1 summarizes market characteristics by study districts, and by block versus village markets. On average, markets operate four days per week and serve a catchment area of approximately eight villages. There is a mean of 68 vendors each market day, however only 12 are female. Nearly two-thirds of markets are privately owned, most commonly by local landlords or business interests, while 16 percent operate without any formal management structure. Markets in Samastipur District are larger and serve smaller geographical areas than in Bhojpur District. Furthermore, block markets operate more frequently and serve twice the number of villages than village markets do.

**Table 1:** Characteristic features of the sample markets.

Market characteristics	Total	District		Market type	
Bhojpur	Samastipur^#^	Block market	Village market^##^
**Market functional factors**					
Number of market operation days	4.0 (2.4)	5.0 (2.5)	3.1 (2.0) ***	4.9 (2.5)	3.9 (2.4) **
Number of villages served by the market	7.6 (8.4)	8.4 (10.5)	7.0 (6.0)	14.6 (17.9)	6.8 (5.9) ***
Number of vendors on a typical day	67.9 (64.6)	56.0 (62.5)	78.3 (64.8) ***	151.5 (104.4)	57.6 (49.1) ***
Number of female vendors on a typical day	12.3 (17.9)	4.2 (8.4)	19.3 (20.9) ***	26.1 (30.3)	10.5 (15.0) ***
Average market fee (INR/vendor/day)	11.8 (14.3)	7.6 (10.3)	15.5 (16.3) ***	19.1 (24.8)	10.9 (12.3)
**Market governance factors**					
1. Private business	64.3%	65.5%	63.2% ***	42.9%	67.0% *
2. Temple committee	12.9%	5.0%	19.9%	17.9%	12.3%
3. Government body	6.3%	5.0%	7.4%	14.3%	5.3%
4. No management	16.5%	24.4%	9.6%	25.0%	15.4%
**Market infrastructure factors**					
Number of physical infrastructure facilities in the market	1.1 (1.3)	1.3 (1.4)	1.0 (1.3) *	2.0 (1.8)	1.0 (1.2) ***
Number of communication facilities in the market	1.2 (0.5)	1.4 (0.6)	1.1 (0.4) ***	1.1 (0.4)	1.2 (0.5)
**Market accessibility factors**					
Vehicular access to the market					
1. Excellent	18.8%	21.0%	16.9%	25.0%	18.1%
2. Good	46.3%	41.2%	50.7%	35.7%	47.6%
3. Moderate	23.9%	29.4%	19.1%	25.0%	23.8%
4. Poor	11.0%	8.4%	13.2%	14.3%	10.6%
Distance to all weather roads (km)	0.4 (1.2)	0.4 (1.2)	0.4 (1.3)	0.3 (1.0)	0.4 (1.3)
Distance to the nearest block headquarters (km)	4.9 (2.8)	5.5 (3.1)	4.3 (2.3) ***	1.1 (1.0)	5.4 (2.5) ***
N	255	119	136	28	227

**Notes:** For continuous variables: Mean (Standard deviation), *p*-value for differences across districts are from Mann–Whitney U test. For factor variables: Percentage, *p*-value for differences across districts are from Pearson’s chi^2^-test. * p < 0.1, ** p < 0.05, *** p < 0.01.

^#^- significance difference between column 3 and column 4; ^##^ - significance difference between column 5 and column 6.

Facilities included in the physical infrastructure count in the market are permanent market structure, raised platforms, permanent roof, closed sewage, garbage disposal place, shelter house, toilet, storage house, cold storage unit, and electricity.

Facilities included in the communication infrastructure count in the market are cellular network, price information communication, and food-related advertisements.

Vehicular access to the market was assessed by asking the respondents to rate how easily vendors could bring food items to sell and consumer could reach to buy in the markets.

The markets in our sample are very poorly equipped with infrastructure (Fig 3). Table 1 shows that the average market in our sample has only around one of the ten key physical infrastructure features considered (such as permanent market structure, raised platforms, permanent roof, closed sewage, garbage disposal place, shelter house, toilet, storage house, cold storage unit, and electricity). Many markets (40 percent) feature none of the ten physical infrastructure facilities, and an additional 30 percent feature only one facility (Figs 3(a)).

**Fig 3:**
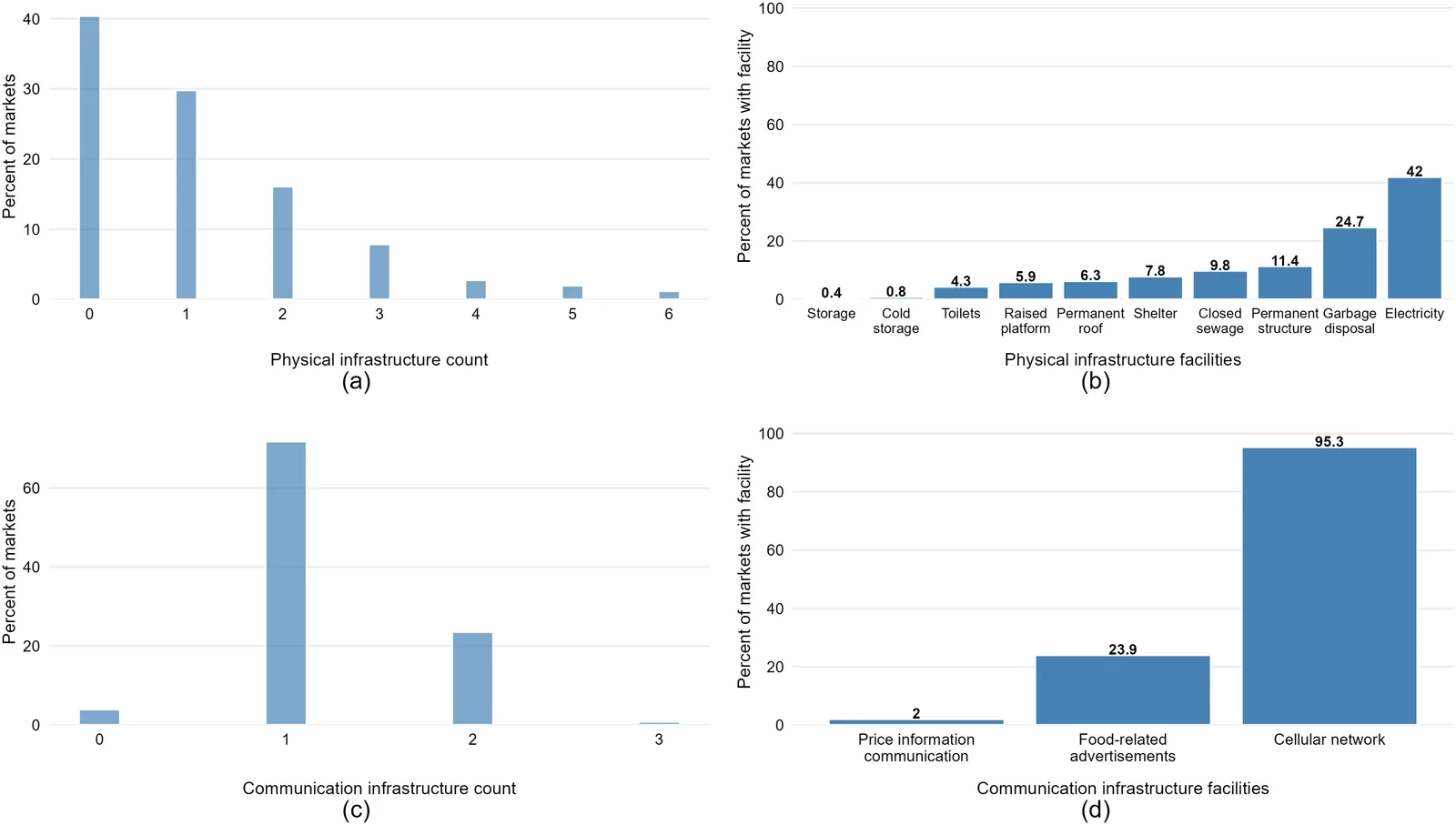
Availability of physical and communication infrastructure facilities in the markets. Notes: (a) Distribution of the number of physical infrastructure facilities in markets. (b) Percentage of markets with or without individual physical infrastructure facilities. (c) Distribution of the number of communication infrastructure facilities in markets. (d) Percentage of markets with or without individual communication infrastructure facilities.

Considering the individual components of physical infrastructure in Fig 3(b), more than 90 percent of markets lack even rudimentary structures such as raised platforms, permanent structures, roofs, and closed sewage. As a result, food has high potential to be exposed to contamination from soil, dust and water from beneath, and direct spoilage from rainfall and sunlight from above, increasing vulnerability to food spoilage, low hygiene and wastage. This is further amplified by the lack of proper waste disposal facilities in about 25 percent of the sample markets (Fig 3(b)), with waste often observed being disposed of along roadsides or in adjacent waterbodies. Furthermore, less than 1 percent of the markets feature any formal cold or non-cold storage facilities, increasing the likelihood of food wastage and lost nutrition potential. Finally, only around 5-7 percent of sample markets have toilet and shelter facilities in the market, rendering market sites less welcoming and convenient, especially for women shoppers and vendors.

Communication technology is important for traders to fix supply arrangements, communicate price movements and advertise produce to potential customers [37]. Whilst 95 percent of markets in Bhojpur and Samastipur districts report reliable mobile phone coverage and/or internet access, only 2 percent of markets have a formal price information system to make food prices available to traders outside the market site (Fig 3(d)). These findings underscore the continued dominance of verbal communication in the markets.

Most of the sample markets have reasonable vehicular access, across block and village markets and study districts (Bhojpur vs. Samastipur). However, about 11 percent of markets have poor vehicular access for vendors and consumers (Table 1). A market on average is about 0.4 kilometres away from an all-weather road and 5 kilometres distance away from the nearest block headquarters.

### 5.2. Market typology

The cluster analysis identifies three market types. While the average silhouette width was highest for the presence of two clusters (S1 Fig), low silhouette width values observed across alternative cluster numbers indicate that the markets in the sample are distributed across a continuum of development rather than representing distinct groups. In this continuum, a large share of markets occupies intermediate positions between two extremes of development levels. This can be confirmed through a visual inspection of the dendrogram (S2 Fig), which supports a three-market cluster solution, with relatively urban and rural markets at the extremes of the continuum and a large number of intermediate markets in the middle. Table 2 presents their characteristics. The first cluster labelled *“emerging urban hubs”*, accounts for 21 percent of the sample markets; 44 percent of these are in the block headquarters with the remaining 56 percent in villages located close to the block headquarters. The second type, *“connected semi-rural markets”*, is the most prevalent market type, comprising 67 percent of markets. Although predominantly village markets, they are in relative proximity to block headquarters and all-weather roads (0.4 and 5.2 kilometres, respectively (Table 2)). The third type, *“remote rural markets”*, constitute 12 percent of the sample and are located farthest (9.2 kilometres) away from the block headquarters in remote rural areas.

**Table 2:** Market characteristic by market types.

Market characteristics	Market type		
Emerging urban hubs^#^	Connected semi-rural markets^##^	Remote rural markets^###^
**Market functional factors**			
Number of market operation days	5.2 (2.4)	3.5 (2.3)	4.4 (2.5) ***
Number of villages served by the market	12.0 (14.1)	6.2 (4.6)	7.7 (9.3) ***
Number of vendors on a typical day	100.4 (92.2)	61.5 (53.5)	45.8 (39.1) ***
Number of female vendors on a typical day	15.7 (24.3)	11.4 (15.5)	10.7 (17.2)
Average market fee (INR/vendor/day)	13.5 (19.8)	11.3 (11.9)	11.5 (15.6)
**Market governance factors**			
1. Private business	51.9%	66.7%	73.3% *
2. Temple committee	9.3%	15.2%	6.7%
3. Government body	9.3%	5.3%	6.7%
4. No management	29.6%	12.9%	13.3%
**Market infrastructure factors**			
Number of physical infrastructure facilities in the market	1.6 (1.5)	0.9 (1.2)	1.3 (1.3) ***
Number of communication facilities in the market	1.2 (0.5)	1.2 (0.5)	1.2 (0.6)
**Market accessibility factors**			
Vehicular access to the market			
1. Excellent	27.8%	17.0%	13.3%
2. Good	48.1%	46.8%	40.0%
3. Moderate	22.2%	23.4%	30.0%
4. Poor	1.9%	12.9%	16.7%
Distance to all weather roads (km)	0.4 (1.1)	0.4 (1.1)	0.8 (1.9)
Distance to the nearest block headquarters (km)	1.5 (1.1)	5.2 (1.8)	9.2 (2.3) ***
N	54	171	30

**Notes:** For continuous variables: Mean (Standard deviation), p-value for differences across market types are from Kruskal-Wallis test. For factor variables: Percentage, *p*-value for differences across districts are from Pearson’s chi^2^-test. * p < 0.1, ** p < 0.05, *** p < 0.01.

^#^- significance difference between column 2 and column 3; ^##^ - significance difference between column 3 and column 4; ^###^ - significance difference between column 4 and column 2.

Facilities included in the physical infrastructure count in the market are permanent market structure, raised platforms, permanent roof, closed sewage, garbage disposal place, shelter house, toilet, storage house, cold storage unit, and electricity.

Facilities included in the communication infrastructure count in the market are cellular network, price information communication, and food-related advertisements.

Vehicular access to the market was assessed by asking the respondents to rate how easily vendors could bring food items to sell and consumer could reach to buy in the markets.

Considering market characteristics, *emerging urban hubs* are significantly larger both in operation (frequency and villages served) and in size than the *connected semi-rural* and *remote rural* markets. Governance patterns remain consistent across market types, with more than two-thirds being privately owned. There is low variation in the presence of different physical and communication infrastructure facilities across market types (Figs 4 and 5). There are minor differences in the total numbers of key physical infrastructural facilities, with *emerging urban markets* exhibiting the highest average (1.6), followed by *remote rural markets* (1.3), and *connected semi-rural markets* (0.9). Furthermore, market connectivity is also similar across market types, with no significant difference in vehicular and all-weather road access to markets (Table 2).

**Fig 4:**
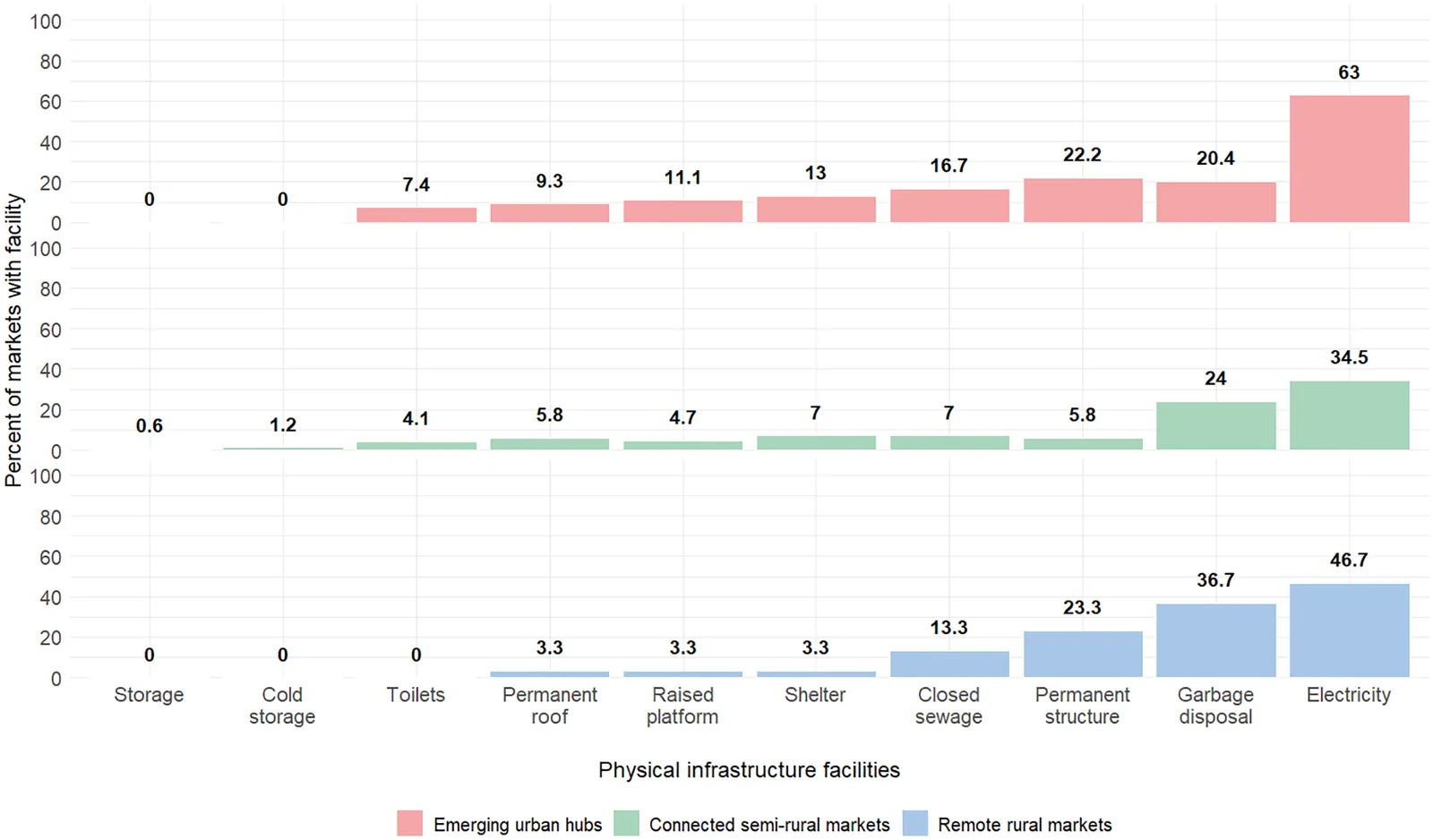
Individual physical infrastructure facilities by market types.

**Fig 5:**
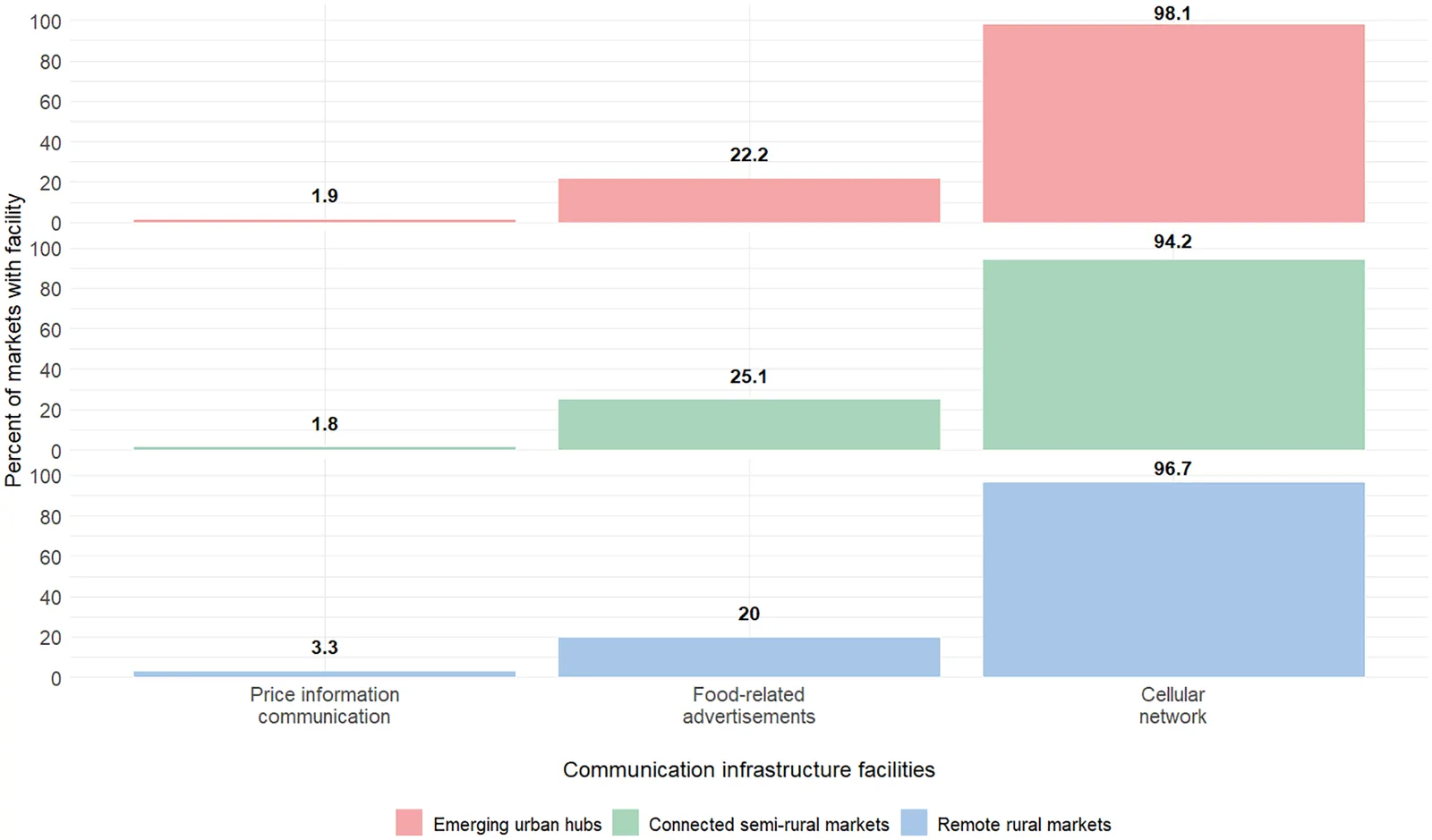
Individual communication infrastructure facilities by market types.

Altogether, despite the identification of three market types, there is remarkably little variation in the multidimensional characteristics of markets in rural Bihar. The only distinguishing aspects are differences in market size and scale of operation, which likely reflect the degree of urbanicity and larger catchment populations in and around block headquarters. This highlights that the rural food markets in our study context represent a continuum, mainly driven by the urbanicity factor, with the majority of markets identified as *connected semi-rural markets* equipped with little to no facilities to enable the availability of diverse, safe, and quality nutrient-dense foods.

### 5.3. Availability and prices of nutrient-dense foods

Do availability and prices of nutrient-dense foods differ across the identified market types, which vary primarily because of the differences in the scale of operation and urbanicity? In Table 3, we present the average number of vendors selling the six different nutrient-dense food groups. Across market types, vegetables are the most available food group in markets. Milk is the least available, reflecting the importance of own production of milk as well as the presence of separate trading arrangements for milk, often sold through separate outlets in villages. Unsurprisingly, given that the market types mainly differ based upon scale of operation and urbanicity, *emerging urban hubs* are found to have significantly higher numbers of vendors across all six food groups than the other two market types (*connected semi-rural* and *remote rural markets*).

**Table 3:** Availability of nutrient-dense foods by market type.

Nutrient-dense foods	Market type		
Emerging urban hubs^#^	Connected semi-rural markets^##^	Remote rural markets^###^
Number of vendors selling vegetables	55.8 (58.4)	40.6 (36.9)	30.9 (28.6) **
Number of vendors selling fruits	12.3 (16.2)	4.8 (8.8)	3.3 (3.9) ***
Number of vendors selling milk products	2.3 (2.9)	0.9 (1.2)	0.7 (1.2) ***
Number of vendors selling eggs	3.5 (4.9)	1.9 (2.0)	2.1 (2.1) *
Number of vendors selling fish	6.2 (6.5)	4.3 (4.5)	3.7 (2.8) *
Number of vendors selling meat	4.1 (4.3)	2.2 (2.0)	1.5 (1.0) ***
N	54	171	30

**Notes:** For continuous variables: Mean (Standard deviation), p-value for differences across market types are from Kruskal-Wallis test. * p < 0.1, ** p < 0.05, *** p < 0.01.

^#^- significance difference between column 2 and column 3; ^##^ - significance difference between column 3 and column 4; ^###^ - significance difference between column 4 and column 2.

In Table 4, we present the average prices of the 17 nutrient-dense foods by market type. While most markets show availability at the food group level (Table 3), the number of markets selling spinach, grapes, papaya, and chicken (local variety) are relatively small, and the reported price averages for these foods must be interpreted with caution.

**Table 4:** Prices of nutrient-dense foods by market type.

Prices of nutrient-dense foods (INR/Kg)	Market type					
N	Emerging urban hubs^#^	N	Connected semi-rural markets^##^	N	Remote rural markets^###^
Tomato	50	61.4 (6.9)	150	63.3 (8.2)	27	61.4 (6.2)
Brinjal	50	37.9 (8.7)	150	35.7 (8.9)	27	34.3 (8.3)
Onion	50	46.1 (3.5)	150	46.0 (2.8)	27	46.5 (4.1)
Potato	49	33.8 (2.4)	151	33.2 (2.1)	27	33.1 (1.9)
Cabbage	39	55.6 (7.9)	86	55.2 (10.0)	13	51.1 (9.2)
Cauliflower	40	88.3 (28.7)	86	89.9 (27.2)	15	76.3 (25.1)
Spinach	15	38.2 (14.5)	25	40.8 (9.2)	1	20.0 (.) *
Spongegourd	50	23.3 (9.6)	150	21.5 (9.0)	26	19.1 (8.1)
Banana	44	49.1 (10.2)	114	45.9 (11.4)	20	46.2 (11.8)
Grapes	1	160.0 (.)	4	127.5 (25.0)	1	160.0 (.)
Jackfruit	24	32.7 (10.0)	80	33.8 (11.4)	15	33.5 (9.3)
Mango	46	69.4 (11.2)	127	63.8 (10.4)	24	60.8 (10.8) ***
Papaya	27	54.2 (8.6)	42	51.8 (11.0)	5	52.5 (7.9)
Egg	39	90.0 (5.9)	100	92.4 (7.1)	15	91.8 (7.8)
Painasa fish	43	144.0 (17.0)	127	146.3 (15.0)	21	148.7 (22.9)
Chicken (broiler variety)	43	188.6 (19.1)	113	189.7 (21.3)	19	200.1 (27.8)
Chicken (desi variety)	8	403.1 (132.6)	14	322.9 (117.7)	1	300.0 (.)

**Notes:** For continuous variables: Mean (Standard deviation), p-value for differences across market types are from Kruskal-Wallis test. * p < 0.1, ** p < 0.05, *** p < 0.01. Standard deviations reported as “(.)” indicate insufficient observations for that specific category.

^#^- significance difference between column 3 and column 5; ^##^ - significance difference between column 5 and column 7; ^###^ - significance difference between column 7 and column 3.

Across market types, Table 4 shows that nutrient-dense foods are generally expensive in comparison to Potato – a staple consumed at almost every meal in Bihar. Table 4 shows the price in Indian Rupees (INR) per kilogram by market type and highlights that the prices are similar across market types. Large, well-connected markets with large numbers of vendors may typically be expected to offer competitive prices. This pattern is observed for some animal source foods such as Painasa fish and broiler chicken, where prices at *emerging urban hubs* are lower than in the other market types. However, Table 4 also shows that, for widely consumed fruits and vegetables such as brinjal (aubergine), sponge gourd, and mango, average prices at *remote rural markets* are lower. For most other fruits and vegetables included in the study, average recorded prices at *remote rural markets* are generally (but not consistently) lower than in the more urban markets. Associations between food prices and market characteristics are presented in S1 Table, which does not show a consistent relationship between prices and any of the market characteristics.

## 6. Discussion

### 6.1. Study implications

Based on a primary survey of 255 rural food markets, our study characterises the landscape of rural markets through which consumers access nutrient-dense foods. We draw three key insights for the development of rural food markets, which have implications for access to healthy foods in India and beyond.

***1. There is a near universal lack of market infrastructure relevant to supporting the availability, affordability, and safety of nutrient-dense foods.***

Our study finds an almost universal and extreme lack of infrastructure and facilities that are important for enabling safe, affordable and equitable access to nutrient-dense foods in Bihar. Rural markets in Bihar fall significantly short of meeting the WHO “healthy markets” guidelines [18,38]. Critical safety and sanitation infrastructure such as waste disposal, wastewater drainage, and the protection of high-risk foods from pests and weather elements such as sunlight and rain is mostly absent. Similarly, our sampled markets do not adhere to the infrastructure standards for “territorial markets” initiative, potentially having adverse implications for the availability, prices, desirability, and safety of nutrient-dense foods sold [17]. While some Indian states (Tamil Nadu, Odisha, Maharashtra, and Uttar Pradesh) display relatively better infrastructure facilities [39,40], others (Meghalaya, West Bengal, and Telangana) show similar challenges observed for markets in Bihar [41,42]. In addition, concerns over the freshness, safety, and hygiene of foods marketed in local food environments are found to influence healthy food acquisition behaviour among consumers [43,44].

Given the complex malnutrition burdens associated with poor diet quality in India, we discuss potential opportunities for Indian Government to improve market conditions. For instance, initiatives such as the “Mahatma Gandhi National Rural Employment Guarantee Act (MGNREGA)” and the National Bank for Rural Development’s (NABARD) “Rural Infrastructure Development Fund (RIDF)” could invest in rural food markets to construct basic infrastructure such as raised platforms, overhead roofs, and waste disposal facilities. This could be complemented with market stakeholder training on good food handling practices to facilitate food safety and prevent food loss [45,46]. From a climate resilience perspective, the almost complete lack of cold storage and shade coverings, combined with the near-absence of drainage facilities and raised platforms, suggests that market places and the foods offered at the markets are vulnerable to both extreme climatic events and multidecadal climate change [12,47]. Additionally, with only one-in-fifty markets connected to a price information system, rural markets and the communities they serve may remain susceptible to climate-related price spikes, owing to the inefficient communication of price information across space. With recent climate projections for Bihar predicting temperature increases >2°C and increasingly erratic precipitation events by 2070 [48], there is an urgent need to expand climate-resilient infrastructure to combat the implications of increased likelihoods and magnitudes of flooding, heatwaves and drought on food availability, prices, and food loss and waste.

***2. Market types differ largely by size and urbanicity but display remarkably little difference in infrastructure and governance.***

Although the cluster analysis identifies three market types, there is only a modest difference in terms of governance and infrastructure facilities. While *emerging urban hub* markets are larger and offer greater availability of nutrient-dense foods, they share similar structural vulnerabilities with rural markets, particularly in terms of exposure to climate shocks, food contamination risks, and food losses. This is in contrast with prior evidence from India showing that administrative remoteness is associated with lower provision of public infrastructure such as roads and schools [49]. In the case of food markets in our sample, however, even those near block headquarters do not appear to be better equipped than remote rural markets. This may reflect the nature of market governance in Bihar, where markets are often privately owned, governed by ultra-local bodies such as GPs or lack formal ownership altogether, as well as limited prioritisation of investments in infrastructure relevant for climate resilience, food safety, and food loss prevention. Although privatisation and globalisation of agri-food markets in LMICs have led to better market facilities in modern supermarkets in urban areas, reflecting rising urban demand for safety and hygiene standards [50], this is not the case in low income settings such as rural Bihar.

The diversity of market ownership or governance models outside the purview of formal state regulation may be the outcome of Bihar government’s repeal of the APMC Act in 2006, which ushered in a new era of minimal state intervention in agricultural marketing. Liberalising the rules governing agricultural trade [51] and the removal of the Act meant an end to the collection of APMC charges, which were designed to be collected from farmers and traders and reinvested into the construction and upkeep of physical infrastructure in the markets [52]. Consequently, any large-scale improvements in market infrastructure, such as the Government of India’s pledge to upgrade 10,000 rural food markets nationwide [53], will need to be led by or at least coordinated alongside a diverse set of stakeholders including landlords, lease holders, market management committees, etc.

***3. Availability of nutrient-dense foods is worse at remote rural markets, but prices are not consistently higher than in emerging urban hubs.***

Despite having better road connectivity and operational size, *emerging urban hub* markets, do not offer consistent price advantages compared to *remote rural* market types. Good transport connections and economies of scale in transport made possible by higher local demand may be expected to lower prices in larger urban markets. On the other hand, previous research [13] has discussed that islands of high prices for perishable foods may form in some remote markets due to high costs of transporting and transacting foods, thus discouraging the flow of foods from other markets in response to price signals.

Our descriptive analysis indicates that, while *remote rural markets* do register higher prices for fish and broiler chicken, most fruit and vegetable prices at *remote rural* markets are not higher than in *emerging urban hubs.* In the case of some locally important nutrient-dense foods such as brinjal (aubergine) and sponge gourd, *remote rural* markets on average have lower prices than urban markets despite their poorer connectivity. This is suggestive of local, short supply chains at play in remote markets that help lower prices. While shorter supply chains have been acknowledged to reduce consumer exposure to external shocks such as natural disasters and economic fluctuations [54,55], strategically developing a mix of both short- and long-distance supply chains can build resilience against disruptions targeting one locality [56,57]. With climate change potentially enhancing the likelihood of local agricultural production failures [58], developing equitable and sustainable transport infrastructure to distribute food, as well as climate-resilient infrastructure to store and protect food at the market, is key to keeping food flowing from surplus to deficit regions [12].

### 6.2. Limitations and future research directions

Some limitations are acknowledged. First, we have presented data from a single point in time, whereas seasonal aspects are recognised as important. While many of the major aspects of our data collection and analysis, such as infrastructure and governance, are largely fixed in time, at least in the medium run, other aspects, such as food availability and prices, are likely to vary seasonally. Furthermore, rapid changes in weather and climatic conditions pose particular challenges to market functioning as well as food availability and prices given the context of limited infrastructure facilities. While our survey was conducted during the peak monsoon season (July and August), at a time of pronounced challenges to market functioning, it is important to acknowledge that availability and prices of foods might differ in other production seasons (e.g., winter or summer). Second, we have assessed nutrient-dense food availability based on the recorded presence or absence of vendors selling each food. While our approach provides insights into the diversity of nutrient-dense foods available in markets, it does not capture volumes. In addition, the price data collected for 17 food items does not account precisely for the differences in their quality within and across the markets. While we acknowledge the importance of accounting for volumes and quality differences in informal food market settings, the complexity and resource-intensity of such data collection warrant further investments in validating proxy-based measures of availability and prices. Third, the study is descriptive and does not claim to undertake any causal inference on market types and food availability and prices. While such causal inference is beyond the scope of this study, future research may seek to disentangle the associations between market characteristics and availability and prices of highly perishable nutrient-dense foods.

The universal lack of market facilities and infrastructure relevant for nutritious diets documented by this study raises the question of the state of food markets in other parts of India and other LMICs. However, apart from a handful of studies of limited geographical scope [59–61], granular data on how well-equipped markets are to support access to nutrient-dense foods are largely missing across the world. In fact, in many countries, even a basic mapping of food market sites beyond large or wholesale markets or government-run markets do not exist. Given the increasing recognition of the central importance of food markets for nutrition [7,62], this represents a glaring deficit in the understanding of how food systems should be transformed to improve nutrition. There is much to gain from a systematic effort to mainstream market-level data collection globally. However, such an ambition must first contend with the challenge that few systematic data collection initiatives currently include a mandate to collect regular data on aspects such as market facilities, infrastructure, governance, availability, and prices.

## 7. Conclusion

This study has collected and descriptively analysed unique granular data on rural food markets in Bihar, India, to ask how well-equipped these markets are to supply poor rural communities with safe and affordable nutrient-dense foods. Concerningly, it finds an extreme and universal lack of basic infrastructure that potentially poses a constraint to the availability and accessibility of nutritious diets to local populations. Markets are found to differ largely by size and urbanicity, but even large, relatively urban markets have no better facilities than remote rural ones. Larger, urban markets offer greater availability of nutrient-dense foods than smaller, remote markets do, but not uniformly lower prices. Modest investments to upgrade rural markets have the potential to boost healthy diets among poor rural populations. Food markets are significantly understudied relative to their importance in nutrition, and a much greater understanding of their functioning in future research is called for.

## Supporting information

S1 Fig Silhouette width from cluster analysis for the market clusters. (PNG)

S2 Fig Dendrogram from cluster analysis summarizing the market types. (PNG)

S1 Table Association between food prices and market characteristics. (DOCX)
