# Supplementary material for: Rural food markets and their implications for the availability and affordability of nutrient-dense foods in Bihar, India
Source: PLoS One. 2026 Oct 30;21(10):e0361231. doi: 10.1371/journal.pone.0361231 (PMC13722213; doi:10.1371/journal.pone.0361231)
Supplement: Supplementary file 1 [file pone.0361231.s003.docx]

|  | Tomato | Brinjal | Onion | Potato | Cabbage | Cauliflower | Spongeg0urd | Banana | Jackfruit | Mango | Egg | Painasa fish | Chicken (broiler variety) |
| --- | --- | --- | --- | --- | --- | --- | --- | --- | --- | --- | --- | --- | --- |
| Market type (ref: 1. Block market) |  |  |  |  |  |  |  |  |  |  |  |  |  |
| 2. Village market | 0.57 | 0.19 | 1.31. | -0.68* | 3.10 | 9.53 | -0.59 | 2.27 | 2.64 | -1.03 | -0.92 | 2.26 | -4.14 |
|  | (1.62) | (1.25) | (0.73) | (0.34) | (2.36) | (8.02) | (0.92) | (2.07) | (2.80) | (2.79) | (1.71) | (4.12) | (6.01) |
| Number of market operation days | 0.14 | 0.22 | 0.19. | 0.12* | -0.48 | 0.88 | 0.28* | 0.11 | 1.30** | 0.72* | -0.67* | -0.73 | 0.09 |
|  | (0.23) | (0.16) | (0.11) | (0.05) | (0.29) | (1.35) | (0.12) | (0.27) | (0.43) | (0.36) | (0.29) | (0.54) | (0.82) |
| Number of villages served by the market | -0.05 | 0.02 | -0.00 | -0.03* | -0.09. | -0.10 | -0.01 | 0.02 | -0.18 | 0.10 | -0.05 | -0.08 | 0.20 |
|  | (0.04) | (0.03) | (0.02) | (0.01) | (0.05) | (0.28) | (0.05) | (0.05) | (0.17) | (0.07) | (0.04) | (0.15) | (0.12) |
| Number of vendors on a typical day | -0.02. | -0.01* | -0.00 | 0.00 | 0.02 | -0.00 | 0.01* | 0.00 | 0.00 | 0.02 | -0.02* | -0.04. | -0.02 |
|  | (0.01) | (0.01) | (0.00) | (0.00) | (0.02) | (0.05) | (0.01) | (0.01) | (0.02) | (0.02) | (0.01) | (0.02) | (0.03) |
| Number of female vendors on a typical day | -0.09** | 0.01 | 0.01 | -0.01 | -0.11. | 0.27 | -0.05** | 0.02 | 0.06 | -0.06 | 0.03 | 0.16 | -0.08 |
|  | (0.03) | (0.02) | (0.02) | (0.01) | (0.07) | (0.19) | (0.02) | (0.04) | (0.05) | (0.06) | (0.04) | (0.11) | (0.13) |
| Average market fee (INR/vendor/day) | -0.04 | -0.02 | 0.00 | -0.01 | -0.02 | 0.15 | -0.05** | -0.00 | 0.02 | -0.08 | 0.07* | 0.41*** | 0.05 |
|  | (0.03) | (0.02) | (0.02) | (0.01) | (0.05) | (0.17) | (0.02) | (0.04) | (0.05) | (0.06) | (0.03) | (0.10) | (0.12) |
| Market governance (ref: 1. Private business) |  |  |  |  |  |  |  |  |  |  |  |  |  |
| 2. Temple committee | 2.66 | 1.06 | 1.18. | -0.08 | 1.25 | 13.43* | -0.20 | 2.25 | 1.28 | 3.12 | -0.75 | 2.87 | 14.82* |
|  | (1.73) | (1.04) | (0.64) | (0.30) | (2.76) | (6.17) | (0.62) | (1.69) | (1.70) | (3.40) | (1.47) | (3.23) | (5.75) |
| 3. Government body | 1.17 | -0.00 | 0.82 | -0.21 | 1.14 | 7.49 | -0.10 | 3.06 | 1.81 | 0.63 | 2.17 | -0.14 | 3.83 |
|  | (1.75) | (1.33) | (0.80) | (0.31) | (2.16) | (9.10) | (0.64) | (1.95) | (3.11) | (2.94) | (1.63) | (5.29) | (6.74) |
| 4. No management | 1.85 | 1.47 | 1.03 | 1.03** | 7.72*** | -4.41 | 1.09 | 1.95 | -0.04 | 1.19 | 3.05 | 7.15. | -5.23 |
|  | (1.34) | (0.96) | (0.67) | (0.39) | (2.12) | (7.83) | (0.70) | (1.51) | (2.78) | (2.33) | (2.01) | (3.85) | (4.99) |
| Number of physical infrastructure facilities in the market | 0.50 | -0.21 | -0.23 | -0.16. | -0.30 | -0.73 | -0.10 | -0.42 | -1.71* | -0.50 | -0.38 | 0.04 | 0.62 |
|  | (0.52) | (0.25) | (0.17) | (0.09) | (0.55) | (2.09) | (0.20) | (0.47) | (0.70) | (0.59) | (0.49) | (0.91) | (1.31) |
| Number of communication infrastructure in the market | -0.45 | -0.63 | 0.29 | 0.13 | -3.28 | -2.28 | -0.61 | -1.29 | -2.19 | 0.51 | 2.76* | 0.43 | 0.48 |
|  | (0.81) | (0.55) | (0.41) | (0.22) | (2.06) | (5.32) | (0.52) | (1.30) | (1.77) | (1.33) | (1.29) | (2.39) | (2.88) |
| Vehicular access to the market (ref: 1. Excellent) |  |  |  |  |  |  |  |  |  |  |  |  |  |
| 2. Good | 1.36 | 0.93 | -0.26 | -0.53* | 2.99 | -7.94 | -0.18 | -6.89*** | -2.30 | -4.95** | 0.24 | 8.10** | 5.73 |
|  | (1.18) | (0.85) | (0.51) | (0.26) | (1.83) | (5.58) | (0.68) | (1.45) | (2.50) | (1.61) | (1.22) | (3.08) | (3.95) |
| 3. Moderate | 1.56 | 0.26 | 0.54 | -0.93** | 1.06 | -8.10 | -0.58 | -6.49*** | -4.56. | -6.13** | 0.27 | 3.77 | 2.37 |
|  | (1.27) | (1.09) | (0.68) | (0.32) | (1.89) | (7.75) | (0.75) | (1.62) | (2.73) | (2.10) | (1.60) | (2.89) | (5.39) |
| 4. Poor | 0.82 | 2.57* | 0.79 | 0.31 | -0.98 | -8.60 | 0.42 | -7.25** | -7.11* | -6.07. | 3.45 | 2.15 | -1.77 |
|  | (1.82) | (1.13) | (0.57) | (0.35) | (3.38) | (8.95) | (0.87) | (2.19) | (3.27) | (3.27) | (2.48) | (5.22) | (5.20) |
| Distance to all-weather road (Km) | -0.77 | -0.61* | 0.22 | -0.01 | -1.38. | -5.10. | -0.46* | -0.04 | -0.62 | 1.92* | -0.66 | -0.45 | 0.73 |
|  | (0.55) | (0.27) | (0.22) | (0.17) | (0.74) | (2.73) | (0.19) | (0.50) | (0.92) | (0.93) | (0.69) | (1.24) | (1.69) |
| Distance to the nearest block headquarters (km) | 0.12 | -0.37* | -0.13 | -0.03 | -0.44 | -1.07 | -0.17 | -0.37 | -0.55 | -0.31 | 0.16 | 0.27 | 1.48. |
|  | (0.17) | (0.15) | (0.12) | (0.04) | (0.27) | (1.04) | (0.12) | (0.25) | (0.36) | (0.33) | (0.20) | (0.48) | (0.83) |
| District (ref: 1. Bhojpur) |  |  |  |  |  |  |  |  |  |  |  |  |  |
| 2. Samastipur | 8.27*** | -14.90*** | -0.04 | -2.46*** | 2.64 | -14.58* | -15.47*** | -17.45*** | -19.82*** | -2.15 | -0.10 | 1.11 | 1.94 |
|  | (1.17) | (0.85) | (0.60) | (0.28) | (2.52) | (6.46) | (0.67) | (1.57) | (3.18) | (1.84) | (1.46) | (2.93) | (4.22) |
| Constant | 58.15*** | 46.05*** | 44.21*** | 35.51*** | 58.58*** | 94.78*** | 32.04*** | 62.40*** | 52.37*** | 67.51*** | 91.66*** | 134.64*** | 180.09*** |
|  | (2.55) | (1.78) | (1.05) | (0.53) | (3.18) | (14.11) | (1.35) | (3.37) | (6.28) | (3.95) | (2.97) | (6.21) | (8.06) |
| N | 227 | 227 | 227 | 227 | 138 | 141 | 226 | 178 | 119 | 197 | 154 | 191 | 175 |

**Notes:** For continuous variables: Standard errors in parentheses. * p<0.1, ** p<0.05, *** p<0.01.

Food prices are measured using Indian Rupees per Kilogram (INR/Kg).

Facilities included in the physical infrastructure count in the market are permanent market structure, raised platforms, permanent roof, closed sewage, garbage disposal place, shelter house, toilet, storage house, cold storage unit, and electricity.

Facilities included in the communication infrastructure count in the market are cellular network, price information communication, and food-related advertisements.

Vehicular access to the market was assessed by asking the respondents to rate how easily vendors could bring food items to sell and consumer could reach to buy in the markets.
