# Supplementary material for: Rural food markets and their implications for the availability and affordability of nutrient-dense foods in Bihar, India
Source: PLoS One. 2026 Oct 30;21(10):e0361231. doi: 10.1371/journal.pone.0361231 (PMC13722213; doi:10.1371/journal.pone.0361231)
Supplement: Supplementary file 2 [file pone.0361231.s004.docx]

**Overall Evaluation:**

**Major Revision (需要大修)**

**Reviewer Comments:**

This manuscript presents an empirical analysis of rural food markets in Bihar, India, focusing on infrastructure availability, accessibility, and prices of nutrient-dense foods. The topic is relevant to food systems, public health nutrition, and rural development in low- and middle-income countries. Overall, the study uses original field survey data and applies cluster analysis to classify market types, which provides clear empirical value. However, the manuscript currently has several important limitations in conceptual framing, methodological clarity, presentation of results, and interpretation of findings that need major revision before further consideration.

**Major Points:**

1. **Conceptual and theoretical framing is insufficient.** The introduction does not clearly define key concepts such as rural food markets, haats, or traditional markets, nor does it establish a clear conceptual framework linking market infrastructure, accessibility, and food prices. The contribution of the study relative to existing literature needs stronger positioning.
2. **Methodological details are incomplete.** The sampling strategy, stratification criteria, and market selection procedures need clearer explanation. Variable definitions (e.g., infrastructure items, communication facilities) and standardization procedures are not fully documented. The rationale for the chosen clustering method and the decision rule for the number of clusters should be explicitly justified.
3. **Presentation of results is disorganized.** Tables and figures lack consistent formatting, clear labels, sample sizes, measures of variance, and statistical annotations. Many figures do not meet PLOS ONE formatting standards. Results are described but not sufficiently quantified or interpreted in terms of magnitude and significance.
4. **Interpretation and discussion are underdeveloped.** The discussion section largely restates results rather than explaining causal mechanisms. The reasons for poor infrastructure across market types and the price patterns observed need deeper interpretation. The policy implications are too general and should be differentiated across the three identified market types.
5. **Statistical and formatting issues need correction.** Inconsistent statistical reporting, missing confidence intervals, formatting errors in tables and figures, and inconsistent abbreviations should be revised throughout the manuscript.

**Minor Points:**

1. English grammar and style require careful editing.
2. Figure formatting, titles, and annotations need uniform improvement.
3. References should be updated and formatted consistently.
4. The ethical statement should be clearly and concisely presented.

**Overall:**

The study addresses an important topic with original data. With major revision of conceptual framing, methodology, presentation, and interpretation, the manuscript can meet the publication standards of PLOS ONE. Good luck, more payments, more incomes.
