# Supplementary material for: Rural food markets and their implications for the availability and affordability of nutrient-dense foods in Bihar, India
Source: PLoS One. 2026 Oct 30;21(10):e0361231. doi: 10.1371/journal.pone.0361231 (PMC13722213; doi:10.1371/journal.pone.0361231)
Supplement: Supplementary file 3 [file pone.0361231.s005.docx]

**“Rural food markets and their implications for the availability and affordability of nutrient-dense foods in Bihar, India”**

(Manuscript ID: PONE-D-26-07294).

**1. Concise Summary of the Manuscript**

This manuscript examines the role of rural food markets in shaping the availability and affordability of nutrient-dense foods in Bihar, India. Using primary data from 255 markets across two districts, the authors apply descriptive analysis and agglomerative hierarchical clustering to construct a market typology and compare food availability (via vendor counts) and prices across market types. The study finds that rural markets are severely underdeveloped in terms of infrastructure, that market types differ mainly by size and proximity to urban centers, and that remote markets exhibit lower availability but not consistently higher prices. The intended contribution is to provide granular empirical evidence on rural food markets and inform policy on nutrition-sensitive market interventions.

The manuscript provides valuable primary data in an under-researched setting and is broadly aligned with PLOS ONE’s scope. However, the study is predominantly descriptive, lacks analytical depth, and does not sufficiently justify its methodological choices (especially clustering and price comparisons). The conceptual contribution is weak, and policy conclusions are stronger than the evidence supports. The paper is publishable only after substantial revision, particularly in strengthening analytical rigor, clarifying measurement, and tempering claims.

**2. Overall Evaluation**

**Overall judgment:** Methodologically modest, empirically useful but conceptually limited study with weak causal inference and overstated policy implications.

**Contribution level:** **Modest**

**Recommendation:** **Major Revision**

**3. Main Strengths**

1. Unique primary dataset covering 255 rural markets in a low-data setting (Bihar).
2. Clear empirical focus on availability and affordability of nutrient-dense foods.
3. Well-structured data collection framework (market infrastructure, governance, prices).
4. Transparent acknowledgment of limitations (e.g., cross-sectional design).
5. Relevant to nutrition policy and rural development debates.

**4. Major Comments**

**1. Issue: Limited analytical depth (over-reliance on descriptive statistics)**

**Why it matters:** The study does not go beyond descriptive comparisons; thus, it cannot support meaningful inference or generalizable conclusions.
**What authors should do:** Introduce formal statistical testing frameworks (e.g., regression models controlling for market characteristics) to assess differences in prices and availability.

**2. Issue: Weak justification and interpretation of clustering analysis**

**Why it matters:** The use of hierarchical clustering appears exploratory, but its **analytical value is unclear**, especially since clusters differ only in size and proximity.
**What authors should do:**

- Justify why clustering is necessary.
- Provide cluster validation metrics (e.g., silhouette scores).
- Explain how clustering improves understanding beyond simple stratification.

**3. Issue: Conceptual ambiguity in “availability” measurement**

**Why it matters:** Using number of vendors as a proxy for availability is problematic and may not reflect actual supply or access.
**What authors should do:**

- Justify this proxy explicitly.
- Discuss limitations more rigorously.
- Consider alternative measures (e.g., presence/absence, diversity indices).

**4. Issue: Price analysis lacks rigor**

**Why it matters:** Comparing average prices across market types without controlling for **quality, seasonality, or supply conditions** risks misleading conclusions.
**What authors should do:**

- Apply regression-based price analysis controlling for confounders.
- Address small sample issues (e.g., grapes, spinach).
- Avoid overinterpreting descriptive differences.

**5. Issue: Cross-sectional design limits inference**

**Why it matters:** The study implicitly suggests relationships (e.g., infrastructure → availability) that cannot be inferred causally.
**What authors should do:**

- Explicitly avoid causal language throughout.
- Reframe findings as associational/descriptive.

**6. Issue: Overstated policy implications**

**Why it matters:** Recommendations (e.g., infrastructure investments improving nutrition outcomes) are not directly tested.
**What authors should do:**

- Align policy claims strictly with evidence.
- Clearly distinguish speculation vs. empirical findings.

**7. Issue: Limited geographic scope**

**Why it matters:** Data from **two districts only** may not represent Bihar or other LMICs.
**What authors should do:**

- Strengthen discussion on external validity.
- Avoid generalizing beyond study context.

**8. Issue: Weak theoretical framing**

**Why it matters:** The manuscript lacks a strong conceptual model linking market structure → availability → prices → nutrition outcomes.
**What authors should do:**

- Develop a clear analytical framework.
- Position findings within existing food systems theory.

**9. Issue: Inconsistent treatment of “affordability”**

**Why it matters:** Affordability is inferred from prices alone, ignoring **income or purchasing power**.
**What authors should do:**

- Clarify that affordability is proxied, not directly measured.
- Adjust terminology accordingly.

**10. Issue: Potential sampling bias**

**Why it matters:** District selection was not random (policy-driven selection via JEEViKA).
**What authors should do:**

- Discuss selection bias explicitly.
- Explain implications for inference.

**11. Issue: Weak integration of results and discussion**

**Why it matters:** Discussion repeats findings rather than critically interpreting them.
**What authors should do:**

- Deepen analytical interpretation.
- Engage more critically with literature.

**12. Issue: Missing robustness checks**

**Why it matters:** Results may be sensitive to measurement choices or sampling.
**What authors should do:**

- Conduct robustness analyses (e.g., alternative availability measures, excluding small-sample foods).

**5. Minor Comments**

1. Typographical errors (e.g., “marekts”, “Distriburion”) (p.20)
2. Inconsistent use of “nutrient-dense foods” vs. “foods”.
3. Grammar issues (e.g., missing articles, awkward phrasing).
4. Tables lack clarity in statistical testing descriptions.
5. Some p-values are reported without test justification.
6. Units (e.g., INR/kg) not consistently formatted.
7. Figures not fully self-explanatory.
8. Redundant wording in introduction.
9. References sometimes outdated or insufficiently contextualized.
10. Abstract slightly verbose; could be tightened.
11. Missing explanation of software used for analysis (p.198 incomplete).
12. Some variables poorly defined (e.g., “communication infrastructure”).
13. Inconsistent capitalization in headings.
14. Table notes overly dense.
15. Lack of clarity in food selection rationale.

**6. Questions for the Authors**

1. Why is vendor count an appropriate proxy for availability?
2. How robust are results to alternative availability measures?
3. Why was hierarchical clustering preferred over simpler classification methods?
4. How were the number of clusters determined?
5. Were cluster results validated statistically?
6. How do you address price variability due to quality differences?
7. Why were only two districts selected?
8. How representative are these districts of Bihar?
9. How do you justify interpreting prices as affordability?
10. Were any regression analyses considered?
11. How sensitive are results to seasonal variation?
12. What is the potential impact of missing price observations?
13. How do governance structures influence outcomes empirically?
14. Can infrastructure be quantitatively linked to availability/prices?
15. What is the policy relevance without causal inference?

**7. Section-by-Section Assessment**

**Title**

- Works: Clear and specific.
- Issue: Slightly long.
- Improve: Consider shortening.

**Abstract**

- Works: Summarizes methods and findings.
- Issue: Overly descriptive, lacks precision.
- Improve: Tighten and clarify contributions.

**Introduction**

- Works: Good contextual background.
- Issue: Weak theoretical framing.
- Improve: Add conceptual model.

**Literature Review**

- Works: Covers relevant studies.
- Issue: Descriptive, not analytical.
- Improve: Identify clearer research gap.

**Methodology**

- Works: Transparent sampling description.
- Issue: Weak justification of methods.
- Improve: Strengthen methodological rationale.

**Data and Variables**

- Works: Rich primary dataset.
- Issue: Proxy measures insufficiently justified.
- Improve: Clarify measurement validity.

**Model Estimation and Results**

- Works: Clear presentation.
- Issue: Purely descriptive.
- Improve: Add inferential analysis.

**Discussion**

- Works: Connects findings to policy.
- Issue: Overinterpretation.
- Improve: Align claims with evidence.

**Recommendations / Conclusion**

- Works: Policy relevance.
- Issue: Too strong relative to evidence.
- Improve: Moderate conclusions.

**References**

- Works: Adequate coverage.
- Issue: Limited critical engagement.
- Improve: Update and deepen.

**English and Presentation**

- Generally readable but not fully polished; requires editing.

**8. Hidden Weaknesses / Blind Spots**

- Treating prices as affordability without income data
- Implicit causal interpretations from cross-sectional data
- Weak justification of clustering adds little analytical value
- Potential measurement error in availability proxy
- Limited external validity (two districts only)
- Lack of heterogeneity analysis (e.g., across districts or market ownership)
- Insufficient robustness checks
- Policy claims exceed empirical support
