# Supplementary material for: Rural food markets and their implications for the availability and affordability of nutrient-dense foods in Bihar, India
Source: PLoS One. 2026 Oct 30;21(10):e0361231. doi: 10.1371/journal.pone.0361231 (PMC13722213; doi:10.1371/journal.pone.0361231)
Supplement: Supplementary file 4 [file pone.0361231.s006.docx]

# ****Review Report****

## ****Summary****

This manuscript examines the role of rural food markets in shaping the availability and affordability of nutrient-dense foods in Bihar, India. Using primary survey data from 255 markets across two districts, the authors analyze market infrastructure, governance, and spatial characteristics, and apply hierarchical clustering to identify market typologies. The study further explores whether food availability and prices vary across market types. The topic is relevant and contributes valuable empirical evidence to an under-researched area in low- and middle-income countries.

## ****Major Comments****

1. **Clarity and positioning of the research contribution**
   The manuscript addresses an important research gap regarding rural food markets in LMICs. However, the novelty of the contribution could be more clearly articulated. While the paper emphasizes “granular data” and “market typologies,” it would benefit from a clearer explanation of how this study advances beyond existing literature, particularly in terms of methodological or policy innovation.
2. **Methodology – need for greater transparency and justification**
   The overall methodological framework is appropriate, but several aspects require clarification:

The **sampling strategy** (stratified random sampling based on distance to block headquarters) is well described; however, the rationale for using Euclidean distance as a proxy for market access and development should be further justified.

The **cluster analysis** (agglomerative hierarchical clustering using Gower distance and Ward’s method) is appropriate, but the manuscript lacks:

- justification for the **selection of variables included in clustering**

- explanation of how the **optimal number of clusters (three)** was determined

The study relies heavily on **descriptive analysis**, which limits interpretability. The authors should discuss why no inferential or econometric methods were used.

3. **Measurement of key variables (availability and prices)**
   There are important limitations in how key variables are operationalized:

**Availability** is proxied by the number of vendors, which may not accurately reflect actual food supply or volume. This limitation is acknowledged but should be discussed more critically in the main analysis section.

**Price data collection**:

- Prices are collected from a maximum of three vendors per market, which may introduce bias

- No discussion of **price variability or dispersion** within markets

- Quality differences across products are not controlled for

4. **Interpretation of results (risk of overgeneralization)**
   Some interpretations appear stronger than what the data can support:

The conclusion that **infrastructure investment could improve nutrition outcomes** is plausible but not directly tested in the study

The discussion linking results to **climate resilience and long-term projections** (e.g., temperature increases by 2070) is interesting but somewhat speculative given the cross-sectional nature of the data

5. **External validity and generalizability**
   The study is based on **two districts in Bihar**, yet some conclusions are framed more broadly (e.g., implications for LMICs or “further afield”).

The authors should more explicitly discuss the **limits of generalizability**

Provide justification for why Bihar can be considered representative (or not)

6. **Ethics and data transparency**
   Ethical approval and consent procedures are clearly stated, which is a strength.
   The data availability statement indicates that all data are included in the manuscript and supporting files, which aligns with journal requirements. However, providing a **public repository link (if possible)** would improve transparency and reproducibility.

## ****Minor Comments****

There are minor language and formatting issues throughout the manuscript (e.g., typos such as “marekts”, spacing inconsistencies).

Figures and tables should be more clearly referenced and explained in the text.

Some recent references (e.g., 2025 citations) should be checked for availability and accuracy.

The description of statistical analysis ends abruptly (“All data curation and analyses presented in the study are done using…”), which should be completed.

Units and formatting in Table 4 (prices) could be standardized for readability.

## ****Recommendation****

☐ Accept
☐ Minor Revision
☑ **Major Revision**
☐ Reject

## ****Justification****

The manuscript addresses an important and underexplored topic and provides valuable primary data. However, significant revisions are required to improve methodological transparency, strengthen the interpretation of results, and clarify the contribution of the study. Addressing these issues would substantially enhance the rigor and impact of the paper.
