# Supplementary material for: Rural food markets and their implications for the availability and affordability of nutrient-dense foods in Bihar, India
Source: PLoS One. 2026 Oct 30;21(10):e0361231. doi: 10.1371/journal.pone.0361231 (PMC13722213; doi:10.1371/journal.pone.0361231)
Supplement: Supplementary file 5 [file pone.0361231.s007.docx]

**Comments to Author(s)**

I reviewed the manuscript entitled *"Rural food markets and their implications for the availability and affordability of nutrient-dense foods in Bihar, India"* (Manuscript ID: PONE-D-26-07294). While the study addresses a relevant topic and the primary data collection is commendable, the manuscript has fundamental deficiencies in analytical rigour, internal consistency, and scholarly presentation that cannot be resolved through revision. I recommend rejection. My specific concerns are as follows.

- The data analysis section is fatally incomplete. Page 12, lines 198–199 end mid-sentence: *"All data curation and analyses presented in the study are done using"* — the software, packages, and version numbers are never stated. In a methods-driven quantitative paper, this omission makes the analysis entirely non-reproducible and unverifiable.
- The manuscript's central claim is internally inconsistent with its own data. The authors state that market types show *"remarkably little difference in infrastructure"* (page 15, lines 273–274), yet Table 2 (page 16) shows remote rural markets score significantly *higher* on physical infrastructure than semi-rural markets (1.3 vs. 0.9, p < 0.01). A paper cannot contradict its own data in the text without explanation.
- The key affordability finding is unsubstantiated. The claim that remote rural prices are *"not consistently higher"* than urban prices (Abstract, lines 28–29) is the paper's most novel contribution, yet Table 4 (pages 18–19) — the sole basis for this claim — reports no significance tests, unlike every other table in the manuscript. An unsupported descriptive comparison cannot carry the weight of a main finding.
- The vendor-count measure of food availability is not validated. The number of vendors is used throughout as a proxy for food availability (page 12, lines 188–190), but no evidence is provided that vendor counts correspond to volumes accessible to consumers. This is a fundamental validity gap for a paper whose core focus is food access.
- Seasonal bias is not adequately addressed. Data collection in July–August (page 22, lines 429–432) coincides with the *kharif* harvest peak—the period of maximum produce availability and lowest prices. The authors acknowledge seasonality as a limitation but do not address how this timing likely inflates availability and deflates prices, rendering both headline findings optimistic estimates.
- The manuscript contains a verifiable factual error. Page 12, line 209 cites "(Figure 1)" in reference to the infrastructure data — Figure 1 is the study area map. The correct reference is Figure 2. This type of basic error signals insufficient proofreading.
- Figure captions contain repeated spelling errors throughout. "*marekts*", "*Distriburion*", "*physcial*", "*infrasturcture*", and "*facilites*" appear across Figure 2, 3, and 4 captions (pages 17, 18, 20). Additional errors include "*precent*" (page 15, line 262) and "*sturdy* districts" in place of "study districts" (page 15, line 365). The volume of such errors across the manuscript is inconsistent with submission to a peer-reviewed journal.
- The manuscript does not conform to PLOS ONE citation style. Author–year in-text citations are used throughout. PLOS ONE requires numbered citations. This indicates the paper was not prepared for this journal.

Taken together, these concerns, an unfinished methods section, a main finding contradicted by the paper's own table, unsupported headline claims, and a volume of presentation errors that suggest the manuscript was not adequately prepared before submission, constitute grounds for rejection rather than revision. I recommend **Rejection.**
