# Supplementary material for: Rural food markets and their implications for the availability and affordability of nutrient-dense foods in Bihar, India
Source: PLoS One. 2026 Oct 30;21(10):e0361231. doi: 10.1371/journal.pone.0361231 (PMC13722213; doi:10.1371/journal.pone.0361231)
Supplement: Supplementary file 6 [file pone.0361231.s008.docx]

Referee Report on:

Rural food markets and their implications for the availability and affordability of nutrient-dense foods in Bihar, India

Manuscript Number: PONE-D-26-07294

This paper studies how market infrastructure, governance, and spatial organization shape the availability and affordability of nutrient-dense foods. By conducting stratified sampling in two districts of Bihar, the study collects data to examine whether the prices of nutrient-dense foods differ by market type. It finds that there is an extreme lack of market infrastructure required for access to safe and affordable nutrient-dense foods. (ii)Market types differ by size and proximity to the nearest urban center, but not much in infrastructure and governance. (iii) Availability of nutrient-dense foods is lower in remote rural markets, but prices in these markets are not consistently higher than in urban markets.

The paper studies an important topic. But I have serious concerns about the depth of the research. I mention some of them here, hoping it will help with improvement. Throughout the paper, I struggled to understand the main contribution and the specific research question they actually addressed (not what they intended to address). Is it the analysis of the physical structure of facilities and access to the rural market? Or is the availability, access (and prices) of nutrient-dense foods the main concern? Or, is it both? If yes, then how does the paper establish a connection between the two? Is the argument that the lack of facilities is a main hindrance to access to nutrition-dense foods? If yes, then my question is, lack of facilities would be a hindrance to any type of food, so why focus on nutrition-dense only? While I accept that nutrition should be the primary focus, I am not sure whether the fact that other foods are readily available indicates that only nutrition is lacking. Even after accepting the noble intention to study nutrient-dense foods, I am not sure that the paper actually addressed what it claims its intention to be.

A similar concern arises when comparing prices between urban and rural markets. Do you intend to focus on the market infrastructure important for nutrient-deficient food access? Probably, taking a more narrow down stand to start with would help the paper. Then the authors could move to extensions.

I think the paper would benefit greatly from a focused research question. E.g., focusing on the climate-resilience perspective to supply nutrient-dense foods would be a valuable contribution. They mention this point somewhere in the text in a more silent tone. But this could bring a lot more value. E.g., without having climate-resilient infrastructure, how would food lose its nutritional value, and how would that be harmful to society, would be a good research question.

One could also focus or engage more in “This suggests that proximity to administrative and economic centres does not necessarily translate into better-equipped food markets,” as written in lines 370-371. With the data in hand, they could estimate the effects of proximity to administrative and economic centers on improvement if better-equipped food markets.

The repeal of the APMC Act in 2006 could be another focus for measuring its impact on at least one of those dimensions. Even with cross-sectional data, one could generate suggestive evidence using the variation on baseline infrastructure and how the repeal of the APMC Act exposed different markets. The administrative data could be combined with the primary survey data for this purpose.

I completely agree with the paper's last line, which concludes that food markets are understudied relative to their importance for nutrition, but the current paper should have focused more on a specific aspect of that area.

Line 456: This study “presented” unique granular data, or “collected/used?”

**Other comments:**

- Thorough English editing required, removing Chat GPT-type punctuation, like unnecessary dashes (e.g., lines 322-323, plus many other places).
- Or for the English language, like in line 162, a sentence ending with “from.”
- Line 198 is an incomplete sentence.
- Line 141-146: Double parentheses within text should be avoided. You do not need to mention reference numbers in the text, as those are already available at the bottom of your manuscript while being published, and available to reviewers in the submitted draft.
- Figure 2 is not readable. Fonts need to be increased.
