# Supplementary material for: Rural food markets and their implications for the availability and affordability of nutrient-dense foods in Bihar, India
Source: PLoS One. 2026 Oct 30;21(10):e0361231. doi: 10.1371/journal.pone.0361231 (PMC13722213; doi:10.1371/journal.pone.0361231)
Supplement: Supplementary file 7 [file pone.0361231.s009.docx]

**Reviewer #6**

*Dear reviewer,*

*Thank you for your comments on the manuscript. Below we provide a detailed description on how we have addressed points raised. For ease of reviewing, we produce your comments below in black font and provide our responses in blue italic font.*

Referee Report on:

Rural food markets and their implications for the availability and affordability of nutrient-dense foods in Bihar, India

Manuscript Number: PONE-D-26-07294

This paper studies how market infrastructure, governance, and spatial organization shape the availability and affordability of nutrient-dense foods. By conducting stratified sampling in two districts of Bihar, the study collects data to examine whether the prices of nutrient-dense foods differ by market type. It finds that there is an extreme lack of market infrastructure required for access to safe and affordable nutrient-dense foods. (ii)Market types differ by size and proximity to the nearest urban center, but not much in infrastructure and governance. (iii) Availability of nutrient-dense foods is lower in remote rural markets, but prices in these markets are not consistently higher than in urban markets.

The paper studies an important topic. But I have serious concerns about the depth of the research. I mention some of them here, hoping it will help with improvement. Throughout the paper, I struggled to understand the main contribution and the specific research question they actually addressed (not what they intended to address). Is it the analysis of the physical structure of facilities and access to the rural market? Or is the availability, access (and prices) of nutrient-dense foods the main concern? Or, is it both? If yes, then how does the paper establish a connection between the two? Is the argument that the lack of facilities is a main hindrance to access to nutrition-dense foods? If yes, then my question is, lack of facilities would be a hindrance to any type of food, so why focus on nutrition-dense only? While I accept that nutrition should be the primary focus, I am not sure whether the fact that other foods are readily available indicates that only nutrition is lacking. Even after accepting the noble intention to study nutrient-dense foods, I am not sure that the paper actually addressed what it claims its intention to be.

*Thank you for this comment. Our study conducts a descriptive and exploratory analysis of market functional, governance, infrastructure, and accessibility characteristics of rural food markets to establish market types. Following this we explore, again using descriptive analysis, how availability and prices of selected nutrient-dense foods vary the established market types. Even though, we have conducted a regression analysis to test the correlations between market characteristics and food prices (Appendix Table A1), this analysis is limited statistical power to establish any meaningful associations. Hence, we do not include them as part of the main outcomes of the manuscript.*

*Coming to the rationale for studying the nutrient-dense foods. While we agree that broader infrastructure facilities will have implications for all kinds of foods sold in the markets, the scale and magnitude of challenges posed by lack/poor physical and communication infrastructure facilities in the markets on nutrient-dense foods like fruits, vegetables, and animal-source foods (as discussed in Section 1, paragraph 3) is much more pronounced, due to their perishability feature. In fact, with rapid urbanisation and retail revolution, food markets and outlets even in remote rural areas now offer packaged foods like chips, biscuits, refined flour, sugar, oil, etc. in large quantities. However, the presence of these other non-perishable foods in the markets does not necessarily mean that there is equivalent ease of access for healthy, but more perishable food options such as fruits, vegetables, and animal-source foods that are crucial for improved dietary and nutrition outcomes.*

*Hence, our paper aims to contribute to the limited but emerging literature on the role of rural informal markets as the key nodes of rural food system transformations by conducting a descriptive exploration of market characteristics to establish market types and exploring how food availability and prices change across these market types. We have now included to a conceptual framework section (Section 2) to highlight these linkages.*

A similar concern arises when comparing prices between urban and rural markets. Do you intend to focus on the market infrastructure important for nutrient-deficient food access? Probably, taking a more narrow down stand to start with would help the paper. Then the authors could move to extensions.

*Thank you for this point. As discussed early in the manuscript, the focus of this study is to better understand the rural markets and compare the food prices over market types but not the between rural and urban markets. The market types identified in our manuscript mare mainly driven by the degree of urbanicity in their location, with markets located closer to a Block Headquarter displaying larger market activity in terms of vendor size. This is an outcome of our study and not the focus. However, we do acknowledge that the rural-urban component has important implications for the prices, as also observed in our manuscript, where despite displaying larger market activities, the emerging urban hub market type does not consistent offer food items at lower prices in comparison to their counterparts in remote-rural markets.*

I think the paper would benefit greatly from a focused research question. E.g., focusing on the climate-resilience perspective to supply nutrient-dense foods would be a valuable contribution. They mention this point somewhere in the text in a more silent tone. But this could bring a lot more value. E.g., without having climate-resilient infrastructure, how would food lose its nutritional value, and how would that be harmful to society, would be a good research question.

*Thank you for suggesting this. We agree that a more nuance approach to study the role of climate resilient infrastructure facilities on market outcomes is interesting and important. However, as you can see from the findings, our sample markets predominantly lack even in providing for basic infrastructure such as raised platforms and roofs, and less than 1 percent of the markets have even non-cold storage facilities. In such contexts, there is very less scope for estimating how the presence/absence of climate resilient infrastructure influences the supply of nutrient-dense foods. However, our findings do highlight the opportunities for investing in updating or developing the new market infrastructure while incorporating the sustainable and equitable factors.*

*Nevertheless, we have subsequent manuscripts in the pipeline that conduct qualitative exploration on the role of climate resilient infrastructure on market level outcomes.*

One could also focus or engage more in “This suggests that proximity to administrative and economic centres does not necessarily translate into better-equipped food markets,” as written in lines 370-371. With the data in hand, they could estimate the effects of proximity to administrative and economic centers on improvement if better-equipped food markets.

*Thank for this pointing this. As mentioned before, due to limited statistical power, we have avoided establishing any associational relationships between market characteristics (such as distance to nearest administrative centres like Block Headquarters) with market infrastructure. Instead, we have included a comprehensive list of market characteristics in the cluster analysis to establish market types. As a result, we do find that urbanicity, measured as the distance to nearest Block Headquarters, is one of the main factors distinguishing the identified market types. However, as we can also see from Table 2, the absolute differences in market infrastructure facilities are not that different across the observed market types, that mainly differ by their urbanicity component. This shows that poor infrastructure facilities is a general problem for our sample markets irrespective of their geographical proximity to the nearest administrative or economic centres.*

The repeal of the APMC Act in 2006 could be another focus for measuring its impact on at least one of those dimensions. Even with cross-sectional data, one could generate suggestive evidence using the variation on baseline infrastructure and how the repeal of the APMC Act exposed different markets. The administrative data could be combined with the primary survey data for this purpose.

*Thank you for this suggestion. While we acknowledge that it would be interesting to study this research question, it is beyond the scope of our descriptive study of rural markets. In addition, we are also limited by data availability to product such experimental evidence.*

I completely agree with the paper's last line, which concludes that food markets are understudied relative to their importance for nutrition, but the current paper should have focused more on a specific aspect of that area.

*As mentioned throughout the manuscript, given the lack of empirical evidence on the status of rural informal food markets, the aim of this study is to establish the context of rural markets in Bihar through descriptive evidence and not provide any focused analysis. However, we do have subsequent manuscripts in the pipeline that conduct focused analysis of market prices and market integration using unique high frequency food price data.*

Line 456: This study “presented” unique granular data, or “collected/used?”

*We have now changed the term presented to used.*

**Other comments:**

- Thorough English editing required, removing Chat GPT-type punctuation, like unnecessary dashes (e.g., lines 322-323, plus many other places).

*We have conducted a thorough review and edit of the entire manuscript. We have also ensure that there is limited use of “–“, which is formatting outcome of the word processor.*

- Or for the English language, like in line 162, a sentence ending with “from.”

*We have now removed the word “from” in this sentence.*

- Line 198 is an incomplete sentence.

*Thanks for pointing this. This sentence has now been completed.*

- Line 141-146: Double parentheses within text should be avoided. You do not need to mention reference numbers in the text, as those are already available at the bottom of your manuscript while being published, and available to reviewers in the submitted draft.

*Thanks for pointing this. We have provided all the ethics related information at the end of the methods section, to accommodate the comments by other reviews. However, we are happy to remove them following the final recommendations from the editor and journal requirements.*

- Figure 2 is not readable. Fonts need to be increased.

*Thanks. We have now updated all figure for better clarity.*
