# Supplementary material for: Rural food markets and their implications for the availability and affordability of nutrient-dense foods in Bihar, India
Source: PLoS One. 2026 Oct 30;21(10):e0361231. doi: 10.1371/journal.pone.0361231 (PMC13722213; doi:10.1371/journal.pone.0361231)
Supplement: Peer review history

# Peer review: Decision Letter 0

**DOI:** 10.1371/journal.pone.0361231.r001

**Authors:** Koushik Chowdhury

31 May 2026

Dear Dr. Purushotham,

Thank you for submitting your manuscript to PLOS ONE. After careful consideration, we feel that it has merit but does not fully meet PLOS ONE’s publication criteria as it currently stands. Therefore, we invite you to submit a revised version of the manuscript that addresses the points raised during the review process.

Thank you for your submission. After editorial assessment, I feel it requires major revisions before consider the manuscript further.

Editor's comments:

1. Strengthen the price analysis by accounting for potential confounding factors (e.g., product quality, market characteristics, and limited sample sizes). Please also avoid causal interpretations, as the cross-sectional design does not support causal inference.
2. Revise Figure 2 to improve clarity and presentation.
3. Clarify the study focus regarding differences between urban and rural markets. A more focused analysis on market infrastructure and access to nutrient-rich foods may strengthen the manuscript. Broader extensions can be considered subsequently.
4. Improve methodological transparency and provide stronger justification for the analytical approach and study design.

Please refer to and download the detailed reviewer comments attached in the submission portal and submit a revised manuscript along with a point-by-point response.

Please submit your revised manuscript by Jul 15 2026 11:59PM. If you will need more time than this to complete your revisions, please reply to this message or contact the journal office at plosone@plos.org. When you're ready to submit your revision, log on to https://www.editorialmanager.com/pone/ and select the 'Submissions Needing Revision' folder to locate your manuscript file.

Please include the following items when submitting your revised manuscript:

- A letter that responds to each point raised by the academic editor and reviewer(s). You should upload this letter as a separate file labeled 'Response to Reviewers'.
- A marked-up copy of your manuscript that highlights changes made to the original version. You should upload this as a separate file labeled 'Revised Manuscript with Track Changes'.
- An unmarked version of your revised paper without tracked changes. You should upload this as a separate file labeled 'Manuscript'.

If you would like to make changes to your financial disclosure, please include your updated statement in your cover letter. Guidelines for resubmitting your figure files are available below the reviewer comments at the end of this letter.

If applicable, we recommend that you deposit your laboratory protocols in protocols.io to enhance the reproducibility of your results. Protocols.io assigns your protocol its own identifier (DOI) so that it can be cited independently in the future. For instructions see: https://journals.plos.org/plosone/s/submission-guidelines#loc-laboratory-protocols. Additionally, PLOS ONE offers an option for publishing peer-reviewed Lab Protocol articles, which describe protocols hosted on protocols.io. Read more information on sharing protocols at https://plos.org/protocols?utm_medium=editorial-email&utm_source=authorletters&utm_campaign=protocols.

As the corresponding author, your ORCID iD is verified in the submission system and will appear in the published article. PLOS supports the use of ORCID, and we encourage all coauthors to register for an ORCID iD and use it as well. Please encourage your coauthors to verify their ORCID iD within the submission system before final acceptance, as unverified ORCID iDs will not appear in the published article. *Only*  the individual author can complete the verification step; PLOS staff *cannot*  verify ORCID iDs on behalf of authors.

We look forward to receiving your revised manuscript.

Kind regards,

Koushik Chowdhury, Ph.D.

Academic Editor

PLOS One

**Journal Requirements:**

When submitting your revision, we need you to address these additional requirements.

1. Please ensure that your manuscript meets PLOS ONE's style requirements, including those for file naming. The PLOS ONE style templates can be found at

https://journals.plos.org/plosone/s/file?id=wjVg/PLOSOne_formatting_sample_main_body.pdf and

https://journals.plos.org/plosone/s/file?id=ba62/PLOSOne_formatting_sample_title_authors_affiliations.pdf

2. Please include a complete copy of PLOS’ questionnaire on inclusivity in global research in your revised manuscript. Our policy for research in this area aims to improve transparency in the reporting of research performed outside of researchers’ own country or community. The policy applies to researchers who have travelled to a different country to conduct research, research with Indigenous populations or their lands, and research on cultural artefacts. The questionnaire can also be requested at the journal’s discretion for any other submissions, even if these conditions are not met. Please find more information on the policy and a link to download a blank copy of the questionnaire here: https://journals.plos.org/plosone/s/best-practices-in-research-reporting. Please upload a completed version of your questionnaire as Supporting Information when you resubmit your manuscript.

3. We note that the grant information you provided in the ‘Funding Information’ and ‘Financial Disclosure’ sections do not match.

When you resubmit, please ensure that you provide the correct grant numbers for the awards you received for your study in the ‘Funding Information’ section.

4. Thank you for stating the following financial disclosure:

“The study received financial support from the Indian Food Systems for Improved Nutrition (INFUSION) project funded by the Bill & Melinda Gates Foundation and the UK Government Foreign, Commonwealth and Development Office (Grant Number: INV- 028942).”

Please state what role the funders took in the study.  If the funders had no role, please state: "The funders had no role in study design, data collection and analysis, decision to publish, or preparation of the manuscript."

If this statement is not correct you must amend it as needed.

Please include this amended Role of Funder statement in your cover letter; we will change the online submission form on your behalf.

5. We note that Figure 1 in your submission contain map images which may be copyrighted. All PLOS content is published under the Creative Commons Attribution License (CC BY 4.0), which means that the manuscript, images, and Supporting Information files will be freely available online, and any third party is permitted to access, download, copy, distribute, and use these materials in any way, even commercially, with proper attribution. For these reasons, we cannot publish previously copyrighted maps or satellite images created using proprietary data, such as Google software (Google Maps, Street View, and Earth). For more information, see our copyright guidelines: http://journals.plos.org/plosone/s/licenses-and-copyright.

We require you to either (1) present written permission from the copyright holder to publish these figures specifically under the CC BY 4.0 license, or (2) remove the figures from your submission:

1. You may seek permission from the original copyright holder of Figure 1 to publish the content specifically under the CC BY 4.0 license.

We recommend that you contact the original copyright holder with the Content Permission Form (http://journals.plos.org/plosone/s/file?id=7c09/content-permission-form.pdf) and the following text:

“I request permission for the open-access journal PLOS ONE to publish XXX under the Creative Commons Attribution License (CCAL) CC BY 4.0 (http://creativecommons.org/licenses/by/4.0/). Please be aware that this license allows unrestricted use and distribution, even commercially, by third parties. Please reply and provide explicit written permission to publish XXX under a CC BY license and complete the attached form.”

Please upload the completed Content Permission Form or other proof of granted permissions as an "Other" file with your submission.

In the figure caption of the copyrighted figure, please include the following text: “Reprinted from [ref] under a CC BY license, with permission from [name of publisher], original copyright [original copyright year].”

2. If you are unable to obtain permission from the original copyright holder to publish these figures under the CC BY 4.0 license or if the copyright holder’s requirements are incompatible with the CC BY 4.0 license, please either i) remove the figure or ii) supply a replacement figure that complies with the CC BY 4.0 license. Please check copyright information on all replacement figures and update the figure caption with source information. If applicable, please specify in the figure caption text when a figure is similar but not identical to the original image and is therefore for illustrative purposes only.

The following resources for replacing copyrighted map figures may be helpful:

USGS National Map Viewer (public domain): http://viewer.nationalmap.gov/viewer/

The Gateway to Astronaut Photography of Earth (public domain): http://eol.jsc.nasa.gov/sseop/clickmap/

Maps at the CIA (public domain): https://www.cia.gov/library/publications/the-world-factbook/index.html and https://www.cia.gov/library/publications/cia-maps-publications/index.html

NASA Earth Observatory (public domain): http://earthobservatory.nasa.gov/

Landsat: http://landsat.visibleearth.nasa.gov/

USGS EROS (Earth Resources Observatory and Science (EROS) Center) (public domain): http://eros.usgs.gov/#

Natural Earth (public domain): http://www.naturalearthdata.com/

6. Please remove your figures from within your manuscript file, leaving only the individual TIFF/EPS image files, uploaded separately. These will be automatically included in the reviewers’ PDF.

7. Please include captions for your Supporting Information files at the end of your manuscript, and update any in-text citations to match accordingly. Please see our Supporting Information guidelines for more information: http://journals.plos.org/plosone/s/supporting-information.

8. If the reviewer comments include a recommendation to cite specific previously published works, please review and evaluate these publications to determine whether they are relevant and should be cited. There is no requirement to cite these works unless the editor has indicated otherwise.

**Additional Editor Comments:**

Thank you for your submission. After editorial assessment, the manuscript requires revision before it can be considered further.

Editor's Comments:

1. Strengthen the price analysis by accounting for potential confounding factors (e.g., product quality, market characteristics, and limited sample sizes). Please also avoid causal interpretations, as the cross-sectional design does not support causal inference.

2. Revise Figure 2 to improve clarity and presentation.

3. Clarify the study focus regarding differences between urban and rural markets. Broader extensions can be considered subsequently.

4. Improve methodological transparency and provide stronger justification for the analytical approach and study design.

Please refer to and download the detailed reviewer comments attached in the submission portal and submit a revised manuscript along with a point-by-point response.

[Note: HTML markup is below. Please do not edit.]

Reviewers' comments:

Reviewer's Responses to Questions

**Comments to the Author**

1. Is the manuscript technically sound, and do the data support the conclusions?

Reviewer #1: Partly

Reviewer #2: Yes

Reviewer #3: No

Reviewer #4: Yes

Reviewer #5: No

Reviewer #6: No

**********

2. Has the statistical analysis been performed appropriately and rigorously?

Reviewer #1: No

Reviewer #2: Yes

Reviewer #3: No

Reviewer #4: Yes

Reviewer #5: No

Reviewer #6: No

**********

3. Have the authors made all data underlying the findings in their manuscript fully available?

The PLOS Data policy

Reviewer #1: Yes

Reviewer #2: Yes

Reviewer #3: Yes

Reviewer #4: Yes

Reviewer #5: No

Reviewer #6: Yes

**********

4. Is the manuscript presented in an intelligible fashion and written in standard English?

Reviewer #1: Yes

Reviewer #2: Yes

Reviewer #3: Yes

Reviewer #4: Yes

Reviewer #5: No

Reviewer #6: No

**********

Reviewer #1: I would like to congratulate the authors on an insightful study. As someone who conducts research in the same geographical area, I recognise the considerable effort required to collect high-quality in-person data.

Here are my comments:

1. line 138-139: Was the same sampling procedure followed for block-level markets? A bit more information on the number of block-level markets existing in these two districts is vital.

2. line 168: Data analysis needs to include the types of statistically analysis that were used such as the use of ttest.

However, the rationality behind using ttest and not ANOVA to compare between 3 clusters in unclear. Comparison between 3 or more groups, requires ANOVA or Kruskal Wallis (depending on the normality), which in this case the authors have used 3 ttest to compare between the groups. Kindly provide the rationale in using ttest and not anova.

3. line 198: sentence incomplete.

4. Graphs 2-4: The graphs could be better. The bars in column graphs could be wider in graph 2. Restrict the y-axis to 100 % in graph 3. I would suggest to use a better visualisation tool to represent the information.

5. The discussion would benefit from reducing repetition, as several sentences convey similar points and could be combined to enhance clarity and conciseness. Example, line 337-345 can be concised.

Reviewer #2: Minor Points:

1.English grammar and style require careful editing.

2.Figure formatting, titles, and annotations need uniform improvement.

3.References should be updated and formatted consistently.

4.The ethical statement should be clearly and concisely presented.

Reviewer #3: The manuscript addresses an important and underexplored topic by providing primary data on rural food markets in Bihar, India. The dataset is valuable and the study has the potential to contribute to the literature on food systems and nutrition in low- and middle-income countries. However, in its current form, the manuscript remains largely descriptive and does not yet meet the level of analytical rigor required for publication.

The primary concern is the limited depth of the statistical analysis. The study relies heavily on descriptive statistics and simple comparisons across market types, without employing inferential or multivariate methods to control for confounding factors. As a result, the conclusions—particularly those related to affordability and policy implications—are not sufficiently supported by the evidence. The use of hierarchical clustering is also insufficiently justified and does not add substantial analytical value, especially given that the resulting clusters differ mainly in scale and proximity rather than in meaningful structural characteristics.

In addition, key concepts require clearer definition and more careful operationalization. For example, “availability” is proxied by the number of vendors, which may not accurately reflect actual supply or consumer access, and “affordability” is inferred from prices alone without considering income or purchasing power. These limitations should be explicitly acknowledged and addressed where possible.

The price analysis would benefit from a more rigorous approach, including controlling for potential confounders such as product quality, market characteristics, and limited sample sizes for certain food items. Without such controls, comparisons across market types remain difficult to interpret. Furthermore, the cross-sectional nature of the data does not allow for causal inference, and the manuscript should avoid any language that implies causality.

The discussion section tends to overinterpret the findings and extends beyond what the data can support, particularly in relation to policy recommendations. These should be more cautiously framed and clearly distinguished from empirically supported conclusions.

Finally, while the manuscript is generally readable, it requires careful language editing to address grammatical errors, typographical issues, and inconsistencies in terminology.

Overall, the manuscript has promise due to its original dataset and relevant research question, but substantial revisions are required. Strengthening the analytical framework, clarifying key concepts, improving methodological justification, and aligning conclusions more closely with the evidence will significantly enhance the quality and credibility of the study.

Reviewer #4: Minor Comments

There are minor language and formatting issues throughout the manuscript (e.g., typos such as “marekts”, spacing inconsistencies).

Figures and tables should be more clearly referenced and explained in the text.

Some recent references (e.g., 2025 citations) should be checked for availability and accuracy.

The description of statistical analysis ends abruptly (“All data curation and analyses presented in the study are done using…”), which should be completed.

Units and formatting in Table 4 (prices) could be standardized for readability.

Reviewer #5: I reviewed the manuscript entitled "Rural food markets and their implications for the availability and affordability of nutrient-dense foods in Bihar, India" (Manuscript ID: PONE-D-26-07294). For detailed comments, please see the attached document

Reviewer #6: This paper studies how market infrastructure, governance, and spatial organization shape the availability and affordability of nutrient-dense foods. By conducting stratified sampling in two districts of Bihar, the study collects data to examine whether the prices of nutrient-dense foods differ by market type. It finds that there is an extreme lack of market infrastructure required for access to safe and affordable nutrient-dense foods. (ii)Market types differ by size and proximity to the nearest urban center, but not much in infrastructure and governance. (iii) Availability of nutrient-dense foods is lower in remote rural markets, but prices in these markets are not consistently higher than in urban markets.

The paper studies an important topic. But I have serious concerns about the depth of the research. I mention some of them here, hoping it will help with improvement. Throughout the paper, I struggled to understand the main contribution and the specific research question they actually addressed (not what they intended to address). Is it the analysis of the physical structure of facilities and access to the rural market? Or is the availability, access (and prices) of nutrient-dense foods the main concern? Or, is it both? If yes, then how does the paper establish a connection between the two? Is the argument that the lack of facilities is a main hindrance to access to nutrition-dense foods? If yes, then my question is, lack of facilities would be a hindrance to any type of food, so why focus on nutrition-dense only? While I accept that nutrition should be the primary focus, I am not sure whether the fact that other foods are readily available indicates that only nutrition is lacking. Even after accepting the noble intention to study nutrient-dense foods, I am not sure that the paper actually addressed what it claims its intention to be.

A similar concern arises when comparing prices between urban and rural markets. Do you intend to focus on the market infrastructure important for nutrient-deficient food access? Probably, taking a more narrow down stand to start with would help the paper. Then the authors could move to extensions.

I think the paper would benefit greatly from a focused research question. E.g., focusing on the climate-resilience perspective to supply nutrient-dense foods would be a valuable contribution. They mention this point somewhere in the text in a more silent tone. But this could bring a lot more value. E.g., without having climate-resilient infrastructure, how would food lose its nutritional value, and how would that be harmful to society, would be a good research question.

One could also focus or engage more in “This suggests that proximity to administrative and economic centres does not necessarily translate into better-equipped food markets,” as written in lines 370-371. With the data in hand, they could estimate the effects of proximity to administrative and economic centers on improvement if better-equipped food markets.

The repeal of the APMC Act in 2006 could be another focus for measuring its impact on at least one of those dimensions. Even with cross-sectional data, one could generate suggestive evidence using the variation on baseline infrastructure and how the repeal of the APMC Act exposed different markets. The administrative data could be combined with the primary survey data for this purpose.

I completely agree with the paper's last line, which concludes that food markets are understudied relative to their importance for nutrition, but the current paper should have focused more on a specific aspect of that area.

Line 456: This study “presented” unique granular data, or “collected/used?”

Other comments:

- Thorough English editing required, removing Chat GPT-type punctuation, like unnecessary dashes (e.g., lines 322-323, plus many other places).

- Or for the English language, like in line 162, a sentence ending with “from.”

- Line 198 is an incomplete sentence.

- Line 141-146: Double parentheses within text should be avoided. You do not need to mention reference numbers in the text, as those are already available at the bottom of your manuscript while being published, and available to reviewers in the submitted draft.

- Figure 2 is not readable. Fonts need to be increased.

**********

what does this mean?). If published, this will include your full peer review and any attached files.

If you choose “no”, your identity will remain anonymous but your review may still be made public.

**Do you want your identity to be public for this peer review?**  For information about this choice, including consent withdrawal, please see our Privacy Policy

Reviewer #1: **Yes:**  Miyola Cia Fernandes

Reviewer #2: **Yes:**  Jianwen Wang

Reviewer #3: No

Reviewer #4: **Yes:**  DOAN BA TOAI

Reviewer #5: No

Reviewer #6: No

**********

[NOTE: If reviewer comments were submitted as an attachment file, they will be attached to this email and accessible via the submission site. Please log into your account, locate the manuscript record, and check for the action link "View Attachments". If this link does not appear, there are no attachment files.]

To ensure your figures meet our technical requirements, please review our figure guidelines: https://journals.plos.org/plosone/s/figures

You may also use PLOS’s free figure tool, NAAS, to help you prepare publication quality figures: https://journals.plos.org/plosone/s/figures#loc-tools-for-figure-preparation.

NAAS will assess whether your figures meet our technical requirements by comparing each figure against our figure specifications.

Attachment Submitted filename: renamed_79a16.docx

Attachment Submitted filename: Rural food.docx

Attachment Submitted filename: Review Report.docx

Attachment Submitted filename: Comments to the Author.docx

Attachment Submitted filename: PONE_30th May2026_Rural Food Market.docx

# Author comment: Author response to Decision Letter 1

**DOI:** 10.1371/journal.pone.0361231.r002

31 Jul 2026

Dear reviewers,

Thank you for your comments. Your comments have helped to improve our manuscript. We have provided detailed responses your reviews on our manuscipt in seperate files and have uploaded them along with manuscript files.

Attachment Submitted filename: Response to Reviewer 6.docx

# Peer review: Decision Letter 1

**DOI:** 10.1371/journal.pone.0361231.r003

**Authors:** Koushik Chowdhury

8 Oct 2026

Rural food markets and their implications for the availability and affordability of nutrient-dense foods in Bihar, India

PONE-D-26-07294R1

Dear Dr. Purushotham,

We’re pleased to inform you that your manuscript has been judged scientifically suitable for publication and will be formally accepted for publication once it meets all outstanding technical requirements.

Within one week, you’ll receive an e-mail detailing the required amendments. When these have been addressed, you’ll receive a formal acceptance letter and your manuscript will be scheduled for publication.

An invoice will be generated when your article is formally accepted. Please note, if your institution has a publishing partnership with PLOS and your article meets the relevant criteria, all or part of your publication costs will be covered. Please make sure your user information is up-to-date by logging into Editorial Manager at Editorial Manager® and clicking the ‘Update My Information' link at the top of the page. For questions related to billing, please contact billing support.

If your institution or institutions have a press office, please notify them about your upcoming paper to help maximize its impact. If they’ll be preparing press materials, please inform our press team as soon as possible -- no later than 48 hours after receiving the formal acceptance. Your manuscript will remain under strict press embargo until 2 pm Eastern Time on the date of publication. For more information, please contact onepress@plos.org.

Kind regards,

Koushik Chowdhury, Ph.D.

Academic Editor

PLOS One

Additional Editor Comments (optional):

The author should check the typos and small language editing throughout the manuscript. Check the headings and the text inside the tables and figures.

Reviewers' comments:

Reviewer's Responses to Questions

**Comments to the Author**

Reviewer #1: All comments have been addressed

**********

2. Is the manuscript technically sound, and do the data support the conclusions?

Reviewer #1: Yes

**********

3. Has the statistical analysis been performed appropriately and rigorously?

Reviewer #1: Yes

**********

4. Have the authors made all data underlying the findings in their manuscript fully available?

The PLOS Data policy

Reviewer #1: Yes

**********

5. Is the manuscript presented in an intelligible fashion and written in standard English?

Reviewer #1: Yes

**********

Reviewer #1: The paper is much more clearer now and the methodology is well structured and easier to follow. All the very best.

**********

what does this mean?). If published, this will include your full peer review and any attached files.

If you choose “no”, your identity will remain anonymous but your review may still be made public.

**Do you want your identity to be public for this peer review?**  For information about this choice, including consent withdrawal, please see our Privacy Policy

Reviewer #1: **Yes:**  Miyola Cia Fernandes

**********

# Editor report: Acceptance letter

**DOI:** 10.1371/journal.pone.0361231.r004

**Authors:** Koushik Chowdhury

PONE-D-26-07294R1

PLOS One

Dear Dr. Purushotham,

I'm pleased to inform you that your manuscript has been deemed suitable for publication in PLOS One. Congratulations! Your manuscript is now being handed over to our production team.

At this stage, our production department will prepare your paper for publication. This includes ensuring the following:

* All references, tables, and figures are properly cited

* All relevant supporting information is included in the manuscript submission,

* There are no issues that prevent the paper from being properly typeset

You will receive further instructions from the production team, including instructions on how to review your proof when it is ready. Please keep in mind that we are working through a large volume of accepted articles, so please give us a few days to review your paper and let you know the next and final steps.

Lastly, if your institution or institutions have a press office, please let them know about your upcoming paper now to help maximize its impact. If they'll be preparing press materials, please inform our press team within the next 48 hours. Your manuscript will remain under strict press embargo until 2 pm Eastern Time on the date of publication. For more information, please contact onepress@plos.org.

You will receive an invoice from PLOS for your publication fee after your manuscript has reached the completed accept phase. If you receive an email requesting payment before acceptance or for any other service, this may be a phishing scheme. Learn how to identify phishing emails and protect your accounts at https://explore.plos.org/phishing.

If we can help with anything else, please email us at customercare@plos.org.

Thank you for submitting your work to PLOS One and supporting open access.

Kind regards,

PLOS One Editorial Office Staff

on behalf of

Dr. Koushik Chowdhury

Academic Editor

PLOS One
